# High‐efficiency genome editing of an extreme thermophile *Thermus thermophilus* using endogenous type I and type III CRISPR‐Cas systems

**DOI:** 10.1002/mlf2.12045

**Published:** 2022-12-07

**Authors:** Jinting Wang, Junwei Wei, Haijuan Li, Yingjun Li

**Affiliations:** ^1^ State Key Laboratory of Agricultural Microbiology and College of Life Science and Technology Huazhong Agricultural University Wuhan China; ^2^ Shenzhen Institute of Nutrition and Health Huazhong Agricultural University Shenzhen China; ^3^ Shenzhen Branch, Guangdong Laboratory for Lingnan Modern Agriculture, Genome Analysis Laboratory of the Ministry of Agriculture and Rural Affairs, Agricultural Genomics Institute at Shenzhen Chinese Academy of Agricultural Sciences Shenzhen China; ^4^ College of Biological and Environmental Engineering Xi'an University Xi'an China

**Keywords:** endogenous CRISPR‐Cas system, genome editing, reporter gene, SOD production, *Thermus thermophilus*

## Abstract

*Thermus thermophilus* is an attractive species in the bioindustry due to its valuable natural products, abundant thermophilic enzymes, and promising fermentation capacities. However, efficient and versatile genome editing tools are not available for this species. In this study, we developed an efficient genome editing tool for *T. thermophilus* HB27 based on its endogenous type I‐B, I‐C, and III‐A/B CRISPR‐Cas systems. First, we systematically characterized the DNA interference capabilities of the different types of the native CRISPR‐Cas systems in *T. thermophilus* HB27. We found that genomic manipulations such as gene deletion, mutation, and in situ tagging could be easily implemented by a series of genome‐editing plasmids carrying an artificial self‐targeting mini‐CRISPR and a donor DNA responsible for the recombinant recovery. We also compared the genome editing efficiency of different CRISPR‐Cas systems and the editing plasmids with donor DNAs of different lengths. Additionally, we developed a reporter gene system for *T. thermophilus* based on a heat‐stable β‐galactosidase gene *TTP0042*, and constructed an engineered strain with a high production capacity of superoxide dismutases by genome modification.

## Impact statement

This is the first systematic study of target interference and genome editing capabilities of endogenous clustered regularly interspaced short palindromic repeats (CRISPR) and CRISPR‐associated (Cas) protein (CRISPR‐Cas) systems of *Thermus thermophilus*. This study developed a high‐efficiency genome editing tool and a reporter gene system and constructed a high superoxide dismutases‐production engineered strain for *T. thermophilus*. Our findings provide a reference for the research and industrial application of extreme thermophiles.

## INTRODUCTION

Extremophiles are a type of microorganisms that can grow and reproduce normally under extreme environmental conditions such as extreme temperature, pH, ion concentration, and radioactivity, and they have evolved promising biological components and metabolic pathways to adapt to these complex environments^1^, ^2^. In recent years, extremophiles have aroused great interest among researchers due to their ability to catalyze chemical reactions under harsh conditions and other industrial application potentials^3^, ^4^, ^5^. Temperature is the main factor affecting the growth of microorganisms, and it directly affects the functions of biomolecules and the integrity of cell structures^6^. In bioindustrial manufacture, high‐temperature processing has many advantages, which makes the study of thermophiles extremely valuable. First, thermophiles can withstand the high‐temperature conditions of the bioreactor, thereby reducing the risk of contamination by mesophilic bacteria. Second, high temperature can also improve the solubility of substrates such as polysaccharides, improve the utilization rate of raw materials, reduce the viscosity of fermentation broth, lessen the pressure of homogenization and aeration, and increase the load of the substrate. In addition, increased temperature allows a high synergistic recovery rate of volatile products through distillation or gas extraction, thereby reducing product inhibition and extending the fermentation period of the culture, ultimately increasing the fermentation yield^7^, ^8^, ^9^. Thermophiles are also considered to be one of the most important sources of thermostable enzymes related to industrial manufacture. Thermostable enzymes can catalyze high‐temperature chemical reactions, which are difficult to achieve with mesophilic enzymes, and these unique properties allow their widespread use in the industry^10^, ^11^. To date, the most successful commercial thermostable enzyme is Taq polymerase isolated from the thermophilic bacterium *Thermus aquaticus* ^12^. Despite the high potential of thermophilic microorganisms, there have been no reports on their practical application as industrial production microorganisms. However, mesophiles have been widely used in industrial production^13^, and one of the major reasons for this is that efficient genome editing tools have been well‐established for many mesophiles, but those for thermophiles are still very poorly developed. The lack of genetic transformation systems, insufficient genome sequence information, and limited screening markers are the main obstacles to the development of genome editing tools for thermophiles. In addition, traditional genome editing based on screening markers and homologous recombination is often time‐consuming, low‐efficiency, and imperfect trace‐free editing method^14^. Therefore, the development of efficient genome editing tools applicable to thermophiles will contribute to promoting the practical application of thermophiles.

The clustered regularly interspaced short palindromic repeats (CRISPR) and CRISPR‐associated (Cas) protein (CRISPR‐Cas) system, as an adaptive immune system, are widely present in bacteria and archaea, and this CRISPR‐Cas system can resist genetic element invasion^15^, ^16^, ^17^. In the past decade or so, research has revealed the diversity of CRISPR‐Cas system structures and mechanisms. Based on the differences between the composition of Cas proteins and effector complexes, the CRISPR‐Cas system is currently divided into two classes (classes 1 and 2), further divided into six types (type I–VI), each of which has its signature *cas* gene, and subdivided into 33 subtypes and several variants^18^, ^19^. The CRISPR‐Cas system exerts its immune function through three main steps, namely, adaptation, CRISPR RNA (crRNA) expression, and interference. At the final interference step, the spacer region of crRNA is paired with the complementary target DNA or RNA sequence, which results in the degradation of the target sequence by the action of Cas nuclease^20^, ^21^, ^22^. This site‐specific nuclease, consisting of crRNA and effector proteins, endows the CRISPR‐Cas system with great potential for genome editing. DNA double‐strand breaks (DSBs) produced by the CRISPR‐Cas system can be repaired through homologous directed repair (HDR) or nonhomologous end joining (NHEJ), and genome editing of target loci can be achieved by artificially designing and providing templates for repair. Currently, the CRISPR‐Cas9 and CRISPR‐Cas12a systems derived from bacteria have been developed into powerful genetic manipulation tools that are widely used in a variety of organisms and human cells^23^, ^24^, ^25^, ^26^.

Genome editing of prokaryotes based on the CRISPR‐Cas system includes two strategies. One is to use an exogenous CRISPR‐Cas system, and the other is to use the CRISPR‐Cas system encoded by the organism. The former introduces a complete set of exogenous CRISPR‐Cas systems into the host cell to achieve genome editing, and the commonly used CRISPR‐Cas9 system falls into this category. However, this strategy does not always work in bacteria and archaea, and the use of exogenous CRISPR‐Cas systems has many limitations. For example, the complex intracellular environment and growth conditions of extremophiles may affect the activity of commonly used CRISPR nucleases. In addition, these exogenous CRISPR nucleases may not be imported into some bacteria and archaea because of their intrinsic proteotoxicity^27^, ^28^. Given the wide distribution of CRISPR‐Cas systems in prokaryotes (in ~40% of bacteria and ~90% of archaea)^19^, a better strategy of genome editing for prokaryotes with active CRISPR‐Cas systems is to utilize their endogenous CRISPR‐Cas systems. For example, the endogenous type I‐A and type III‐B systems of the thermophilic archaeon *Sulfolobus islandicus* have been successfully used for genome editing^29^. The type I‐B CRISPR‐Cas system of the haloarchaeon *Haloarcula hispanica* and the type II‐A CRISPR‐Cas system of lactic acid‐producing bacterium *Pediococcus acidilactici* are both highly efficient in performing genome editing^30^, ^31^, ^32^. In addition, multiple types of endogenous CRISPR‐Cas systems have been employed to edit different species with high efficiency, including the type I (I‐A, I‐B, I‐C, I‐E, I‐F, I‐G)^33^, ^34^, ^35^, ^36^, ^37^, ^38^, type II‐A^39^, ^40^, and III‐A^41^.

*Thermus thermophilus* is an extremely thermophilic Gram‐positive bacterium. Some of its properties such as high growth rates, high cell yields of the cultures, and certain valuable natural products such as superoxide dismutase (SOD) make it an excellent model bacterium to study the molecular basis of thermophiles^42^. *T. thermophilus* HB27 was originally isolated from a natural volcanic hot spring in Japan^43^, and its complete genome sequence was reported as early as 2004^44^. In 2016, Godde et al.^45^ reported the presence of CRISPR repeat sequences and *cas* genes on the megaplasmid of *T. thermophilus* HB27, which was confirmed by other researchers who demonstrated the specific distribution of CRISPR and *cas* genes on the megaplasmid and chromosome of the homologous strain *T. thermophilus* HB8^46^. The *T. thermophilus* genome encodes abundant CRISPR‐Cas systems, but at present, genome editing strategies for *T. thermophilus* are still mainly based on a traditional combination of screening markers and homologous recombination^14^. This traditional genome editing method is less efficient, time‐consuming, and highly dependent on the marker used for screening. Thus, it is far from perfect. Recently, there was one reported case of genome editing of *T. thermophilus* HB27 based on the CRISPR‐Cas system, in which a thermostable exogenous Cas9 (CaldoCas9) protein was utilized^47^. Although it could successfully achieve genome editing, the activity of the CaldoCas9 protein was not high at the optimum growth temperature of *T. thermophilus* of 65°C, and the transformation efficiency of the plasmid was low, which might be due to the burden or toxicity of the large molecular weight of Cas9 protein to host. Considering the abundant CRISPR‐Cas systems encoded by *T. thermophilus* HB27, an endogenous CRISPR‐Cas system‐based genome editing strategy is worth developing. In this study, we systematically characterized the target interference capabilities of the endogenous I‐B, I‐C, and III‐A/B CRISPR‐Cas systems encoded by *T. thermophilus* HB27. We developed efficient genome editing tools based on the interference activity of its endogenous CRISPR‐Cas systems, through which genomic manipulations such as genomic deletion, mutation, and in situ tagging could be easily implemented. We also found that the editing efficiency of type I‐B and I‐C CRISPR‐Cas systems was higher than that of type III‐A/B systems. In addition, we constructed the reporter gene system for *T. thermophilus* HB27 using this genome editing tool, which is of great significance for studying promoter activity and gene expression patterns and for identifying transcription factors. Finally, we constructed an engineered strain with a high SOD production capability by genome modification.

## RESULTS

### High‐throughput sequencing analysis of endogenous CRISPR‐Cas systems of *T. thermophilus* HB27

**Figure 1:**
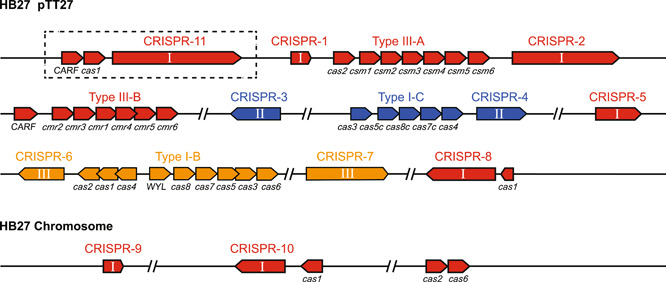
CRISPR‐Cas systems of *Thermus thermophilus* HB27. CRISPR‐Cas loci located on the megaplasmid and chromosome are shown in different colors (yellow for I‐B, blue for I‐C, and red for III‐A and III‐B). CRISPR arrays are numbered and classified according to their repeat types (I, II, and III represent CRISPR arrays belonging to the type III, I‐C, and I‐B CRISPR‐Cas systems, respectively). The sequence in the dotted box indicates the inserted 10 kb fragment. The direction of transcription is indicated by the arrows.

*T. thermophilus* HB27 (GenBank: AE017221.1 and AE017222.1) is the model organism used in this study. Its complete genome sequence was resolved in 2004^44^. The genome of HB27 consists of a circular chromosome (~1.9 Mb) and an endogenous megaplasmid pTT27 (~232 kb). Endogenous type I‐B, I‐C, III‐A, and III‐B CRISPR‐Cas systems are encoded on its megaplasmid, and the genome contains 10 CRISPR arrays falling into three different types of repeats, of which III‐A and III‐B subtypes share the same CRISPR arrays^48^. Although the full genome sequence of HB27 has long been resolved, a recent study has revealed that the CRISPR‐Cas system of HB27 may have some discrepancies from the results previously reported in the database^49^. Considering the reported variability of the *T. thermophilus* strains^50^, we performed whole genome sequencing of the HB27 strain preserved in our laboratory. The sequencing results were consistent with the recent report, the sequence information had a high resemblance to the previously published HB27 genome information^49^, and the only difference in the CRISPR‐Cas system was reflected in the presence of an approximately 10 kb fragment insertion in the upstream sequence at the type III‐A system locus (shown in the dotted boxes in Figure 1). This 10 kb fragment contained a new CRISPR array (CRISPR‐11) belonging to the type III system with 17 spacers. In addition, this 10 kb fragment also encoded two CRISPR‐associated proteins including one Cas1 protein and one protein containing the CARF (CRISPR‐associated Rossmann fold) domain^51^. Moreover, we observed that CRISPR‐11 and CRISPR‐1 shared the same repeat feature, that these two arrays were separated by two copies of transposases, and that the CRISPR‐1 lacked the leader sequence. Based on this observation, we speculated that CRISPR‐1 and CRISPR‐11 might originally belong to the same CRISPR array and that the transposition event‐mediated sequence insertion might lead to the formation of these two CRISPR arrays. All sequence information on the CRISPR‐Cas system in the *T. thermophilus* HB27 genome is shown in Figure 1. The CRISPR repeat sequences and the subtypes to which they belong are listed in Table 1.

**Table 1:** CRISPR repeat sequences of *Thermus thermophilus* HB27.

Repeat sequence	Length (bp)	CRISPR array	CRISPR subtype
GTTGCAAACCTCGTTAGCCTCGTAGAGGATTGAAAC	36	6, 7	I‐B
GTTGCACCGGCCCGAAAGGGCCGGTGAGGATTGAAAC	37	3, 4	I‐C
GTTGCAAGGGATTGARCCCCGTAAGGGGATTGCGAC	36	1, 2, 5, 8, 9, 10, 11	IIIA, IIIB

### Functional identification of different types of endogenous CRISPR‐Cas systems

In this study, we found that *T. thermophilus* HB27 encoded four subtypes of CRISPR‐Cas systems, and the type III‐A and III‐B complexes encoded by its highly homologous strain HB8 have been reported to cleave target RNA and DNA in vitro^52^, ^53^, ^54^. However, the in vivo interference activities of the four subtypes of CRISPR‐Cas systems remain largely unknown. Previous bioinformatics studies found that the type I‐C system of HB27 preferred TTC as the protospacer‐adjacent motif (PAM)^48^, while type I‐B systems generally have a consistent preference for the TTN (N corresponds to A, T, G, or C) PAM sequence^36^, ^55^, ^56^, ^57^. We constructed target plasmids (pRKP31‐IB/IC‐CRSP1‐TTN) carrying a protospacer with different PAM sequences (TTA, TTT, TTC, and TTG) to examine the interference activities and PAM preference of the type I‐B and I‐C CRISPR‐Cas systems in *T. thermophilus* HB27 (Figure S1A). The results revealed that all target plasmids showed lower transformation efficiencies than the control empty plasmid pRKP31, and both type I‐B and I‐C systems possessed good interference activities with the TTC PAM sequence (Figure S1B,C).

**Figure 2:**
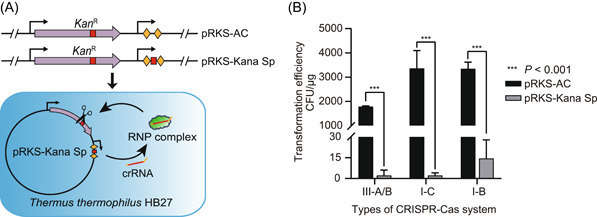
Functional identification of different types of endogenous CRISPR‐Cas systems. (A) Schematic of in vivo interference activity validation using the self‐targeting plasmids. The diamond blocks represent different repeat sequences. The red square represents the spacer sequence designed based on the target. (B) Transformation efficiency of the empty control plasmid (with a nontargeting spacer) and different types of self‐targeting plasmids. AC, artificial mini‐CRISPR; *Kan* ^R^, kanamycin resistant gene; RNP complex, ribonucleoprotein (RNP) complex from CRISPR‐Cas systems. Error bars indicate the standard deviation of three independent replicates.

Next, we constructed a self‐targeting plasmid (pRKS‐Kana sp) carrying an artificial mini‐CRISPR to further examine and compare the interference activities of the endogenous I‐B/C and III‐A/B CRISPR‐Cas systems. Briefly, the spacer of the mini‐CRISPR array carried by the self‐targeting plasmid was designed based on the kanamycin resistance gene on the plasmid. Once the self‐targeting plasmids were transformed into HB27 cells, these plasmids would express crRNAs, thus driving the endogenous CRISPR‐Cas system to disrupt the target site (Figure 2A). If the endogenous CRISPR‐Cas system was active, the resistant gene would be disrupted, resulting in few or no transformants on kanamycin plates. Since the type III system requires no specific PAM sequence, we only needed to ensure the mismatching between the 5′‐end sequence of crRNA and the 3′‐end flanking sequence of the target RNA to activate the DNA cleavage activity of the type III system^22^. It should be noted that the identical repeat sequence shared by the type III‐A and III‐B systems of HB27 prevented us from distinguishing the activity of the III‐A and III‐B systems, and thus, we integrated them into III‐A/B. For type I‐B and I‐C systems, we chose TTC as their PAM sequence. It was shown that the transformation efficiency of the self‐targeting plasmid pRKS‐Kana Sp was significantly lower than that of the control plasmid pRKS‐AC and that transformation efficiency mediated by I‐B, I‐C, and III‐A/B systems was reduced by over 99.5% (Figure 2B), indicating that the endogenous type I‐B, I‐C, and III‐A/B CRISPR‐Cas systems of *T. thermophilus* HB27 all had interference activity for artificial mini‐CRISPR arrays.

### Gene knockout based on different types of endogenous CRISPR‐Cas systems

**Figure 3:**
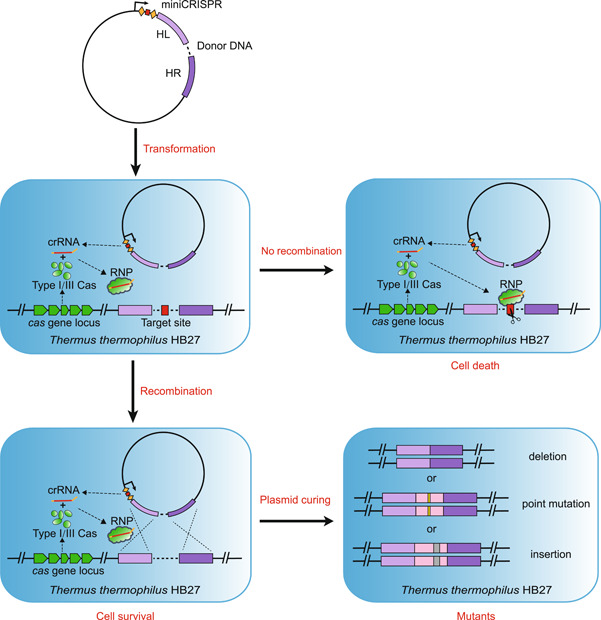
Scheme of genome editing based on different endogenous CRISPR‐Cas systems. Transformation of editing plasmids carrying the repeat–spacer–repeat expression cassette leads to the cleavage of the target region matching with the spacer. Homologous recombination repair by the host with the donor fragment leads to the production of the desired mutant. Strains undergoing no recombination are still able to be cleaved by the CRISPR‐Cas system, and thus, they do not survive. HL and HR indicate the left arm and right arm of the homologous arm, respectively. CRISPR‐Cas, clustered regularly interspaced short palindromic repeat (CRISPR) and CRISPR‐associated (Cas) protein.

As mentioned above, we have verified that the endogenous CRISPR‐Cas systems of *T. thermophilus* HB27 could actively interfere with self‐targeting plasmids to reduce colony‐forming units (CFUs) by three orders of magnitude (Figure 2B). Therefore, these CRISPR‐Cas systems can be developed into gene editing tools, and the specific design scheme is shown in Figure 3. Specific mutations can be introduced by simply adding donor fragments to different types of interference plasmids for homologous recombination repair. In principle, wild‐type cells will die from genomic breaks mediated by the CRISPR‐Cas system, while mutant cells will survive due to mutations at the target loci, thus resulting in the screening and enrichment of desired mutants.

**Figure 4:**
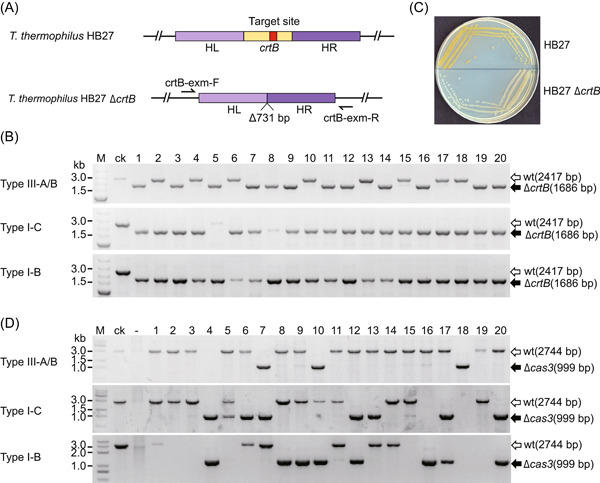
Gene knockout based on different types of endogenous CRISPR‐Cas systems. (A) Sketch of the gene locus comparison between the wild‐type HB27 strain and *crtB* knockout strain. crtB‐exm‐F/crtB‐exm‐R are primers used to verify the target gene deletion. (B) Amplified *crtB* gene locus of the randomly selected colonies with the editing plasmids. The PCR products were verified through agarose gel analysis. (C) Phenotype of the wild‐type HB27 strain and *crtB* knockout strain on the TB plate. (D) Amplified *cas3* gene locus of the randomly selected colonies with the editing plasmids. PCR products were verified through agarose gel analysis. ck, control group with the wild‐type strain as the template; M, DNA marker; wt, wild type.

To investigate the feasibility of genome editing in *T. thermophilus* HB27, we first validated the feasibility of gene knockout based on its endogenous I‐B, I‐C, and IIIA/B systems. *T. thermophilus* HB27 can produce natural pigments, which results in yellow colonies on the TB medium. The *crtB* gene located on the megaplasmid pTT27 was selected as the target gene. This target gene encodes phytoene synthase, which is the first synthase in the carotenoid biosynthetic pathway, and its deletion will disrupt the synthesis of carotenoids, resulting in a change from a yellow to a white colony phenotype of *T. thermophilus* HB27^58^. Three editing plasmids were constructed based on type I‐B, I‐C, and IIIA/B systems for knocking out the *crtB* gene, and the constructed editing plasmids contained a donor DNA fragment consisting of the upstream sequence (HL, ~700 bp) and the downstream sequence (HR, ~700 bp) of the *crtB* gene (Figure 4A). After the transformation of the editing plasmid into *T. thermophilus* HB27, both yellow and white colonies were observed on the plate. Polymerase chain reaction (PCR) validation of randomly selected 20 monoclonals showed that the knockout efficiency was 60% (12/20) for the type III‐A/B system, 95% (19/20) for the I‐C system, and 100% (20/20) for the I‐B system (Figure 4B). As expected, the color of the wild‐type colonies was dark yellow, while the color of the Δ*crtB* colonies was yellowish white (Figure 4C). To verify the universality of this knockout method, we selected another gene (*cas3* gene of the type I‐C system) for knockout verification. We easily obtained the desirable knockout strain by this method (Figure 4D). PCR sequencing further verified that the deletion was introduced in the desired gene locus (Figure S2). The overall knockout efficiency of the *cas3* gene was lower than that of the *crtB* gene, which might be related to the size of the knockout fragment, the length of the homologous arm, and the characteristics of the target gene. We also found that the editing plasmid could be easily cured by a simple screening in the antibiotic‐free medium, which made the iterative genome editing of *T. thermophilus* HB27 quite efficient and convenient.

### Optimization of donor length for improving editing efficiency

**Figure 5:**
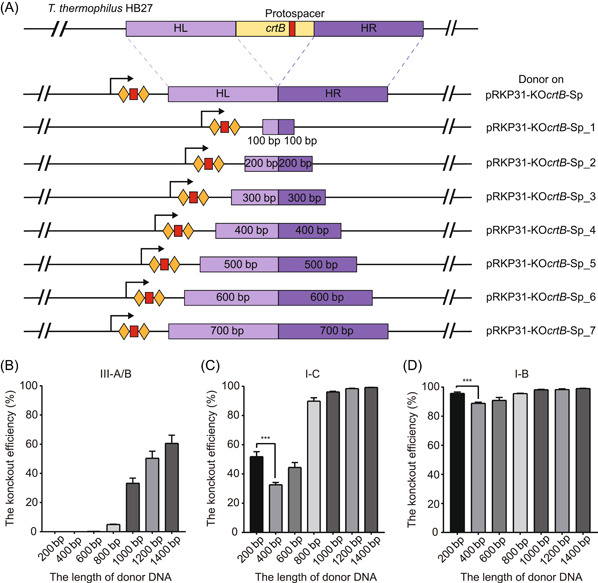
Impact of donor size on gene deletion efficiency. (A) Different donor lengths on pRKP31‐KO*crtB*‐Sp. (B–D) Effect of donor length on *crtB* knockout efficiency of the type III‐A/B CRISPR‐Cas system (B), type I‐C CRISPR‐Cas system (C), and type I‐B CRISPR‐Cas system (D). The knockout efficiency of *crtB* was calculated at the proportion of white colonies on the plates. Error bars indicate the standard deviation (SD) of three independent replicates. ***p < 0.001.

Theoretically, the efficiency of homologous recombination will be improved when the length of the donor is increased, but a very long donor sequence is not conducive to plasmid construction, and it may increase the burden on the host strain. Therefore, it is important to choose a donor sequence with the appropriate length. We designed a series of editing plasmids with different donor lengths (200, 400, 600, 800, 1000, 1200, and 1400 bp) with the *crtB* gene as a knockout target to determine the shortest donor length required to achieve efficient gene editing (Figure 5A). Disruption of the *crtB* gene will result in a change in colony color from yellow to white, and thus, we can determine the knockout efficiency of this editing plasmid by calculating the percentage of white colonies in the total number of colonies. For the type III‐A/B system, the editing efficiency reached a maximum of 60.5% when the donor length was 1400 bp, dropped to 5% when the donor length was shortened to 800 bp, and to 0% for shorter than 800 bp (Figure 5B). The knockout efficiency of the type I‐C system was stable at above 90% when the donor length was more than 800 bp, and the knockout efficiency dropped abruptly to 44% when the donor length was 600 bp. However, the knockout efficiency increased to 51% for a donor length of 200 bp, which was higher than that in the case of the 400 bp donor and 600 bp donor (Figure 5C). We speculated that as the donor length dropped to 200 bp, the donor was not long enough for homologous recombination repair, resulting in large fragment deletions around the *crtB* gene that could also lead to the appearance of white colonies. We hypothesized that the occurrence of this unknown deletion near the target site resulted in increased editing efficiency of the 200 bp donor in type I CRISPR‐Cas systems. PCR sequencing of DNA from white colonies showed a larger proportion of unknown‐size deletions near the target site of the 200 bp donor than the other‐length donors (Figure S4). As the donor length was shortened, the efficiency of accurate deletion decreased so that the efficiency of homologous recombination was reduced. For the type I‐B system, the knockout efficiency of editing plasmids containing 200–1400 bp donor lengths was above 88.9%. Similar to the type I‐C system, the knockout efficiency of the edited plasmid containing a 200 bp donor in the type I‐B system was quite high (Figure 5D), and the reason for this result might be the same as for the type I‐C system mentioned above.

Overall, the knockout efficiency increased with increasing donor length; 1000–1200 bp donor length was sufficient for repairing DNA breaks generated by all four types of CRISPR‐Cas systems and getting the editing efficiency above 50%. The editing efficiency of the type I‐B system was the highest, while that of the type III‐A/B system was the lowest. Notably, the transformation efficiency of editing plasmids based on type III‐A/B systems was significantly higher than that based on type I‐B and I‐C systems (Figure S3). The type III CRISPR‐Cas immunity requires the transcription of the target gene^59^. The higher transformation efficiency and lower editing efficiency are probably due to the relatively low interference activity of type III‐A/B systems because of the low transcription of the *crtB* gene in the polyploid *T. thermophilus* ^60^.

### Gene point mutations based on different types of endogenous CRISPR‐Cas systems

**Figure 6:**
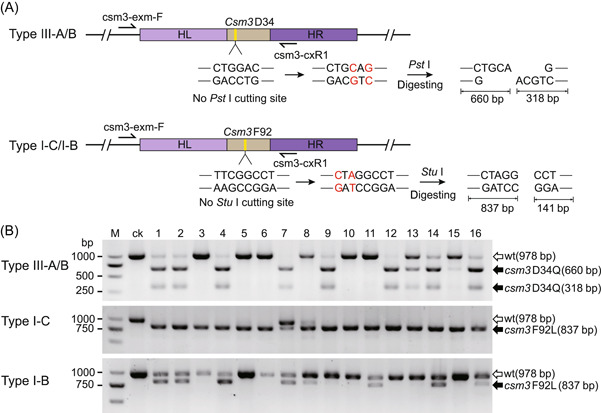
Gene point mutations based on different types of endogenous CRISPR‐Cas systems. (A) Schematic of point mutation detection. The PCR amplification product of the wild‐type *csm3* gene was not digested by *Pst* I or *Stu* I restriction endonuclease. However, the PCR amplification product with point mutation was digested by the restriction endonuclease to obtain two DNA fragments. (B) PCR products of the *csm3* gene amplified from 16 randomly selected transformants (lanes 1 to 16) were digested by *Pst* I or *Stu* I and analyzed with agarose gel. The *csm3* gene of the wild‐type strain was used as the amplification template. ck, control group; M, DNA marker; wt, wild type.

Gene point mutation techniques are important for the study of the gene function and protein active sites. Therefore, we explored the application of this endogenous CRISPR‐Cas system‐based editing tool in gene point mutations. Theoretically, the type III‐A CRISPR‐Cas system derived from *T. thermophilus* should be thermally stable, and the Csm3 protein is the effector protein responsible for the specific cleavage of target RNAs. The aspartate (Asp) at position 34 of the Csm3 protein is essential for its cleavage activity^61^. We mutated this Asp into glutamine (Gln) while also introducing the nucleic acid sequence of Asp into the a *Pst* I restriction site to facilitate our detection of point mutations (Figure 6A). Since the sequence adjacent to the mutation site contained no appropriate PAM sequence for the type I system, we used the type III‐A/B system for editing. To further test the ability of the type I‐B and I‐C systems to perform point mutations, we selected another site F92 on this *csm3* gene for point mutation, and the mutation introduced the *Stu* I restriction site (Figure 6A). After the transformants were obtained, PCR was performed to amplify the fragment near the target site, and the PCR products were digested using the corresponding restriction endonucleases, followed by agarose gel electrophoresis. If the target site has been successfully mutated, the band size of the PCR product after digestion would change. The restriction enzyme digestion results showed that the efficiency of point mutation mediated by the type III‐A/B system was 56.3% (9/16), but wild‐type bands were still present in most mutants (8/9), indicating the presence of a mixed genotype (Figure 6B). The efficiency of point mutations mediated by the type I‐C system was 100% (16/16), and only one single colony had a mixed genotype (Figure 6B). The efficiency of point mutation mediated by the type I‐B system was 50% (8/16), and all mutants still showed wild‐type bands (Figure 6B). PCR sequencing further verified that the mutation was introduced to the desired gene locus (Figure S5A,B). We also performed other forms of single‐nucleotide conversions and the target mutant strains could also be obtained (Figure S5C, S5D). These results indicated that point mutations could be easily achieved based on different types of endogenous CRISPR‐Cas systems. The presence of the mixed genotype of mutants might be related to the fact that *T. thermophilus* is a polyploid bacterium^60^.

### In situ gene tagging based on different types of endogenous CRISPR‐Cas systems

**Figure 7:**
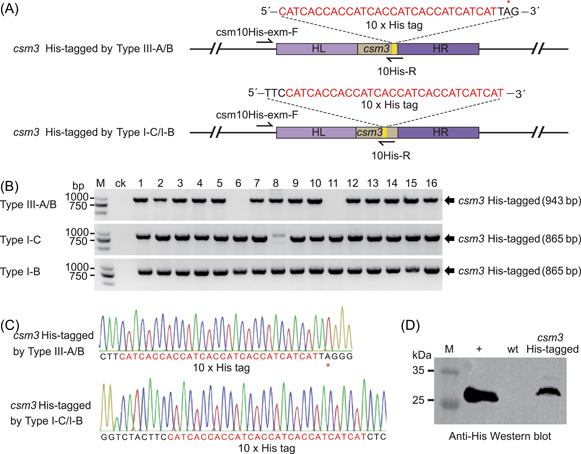
CRISPR‐based in situ gene tagging. (A) Schematic of in situ tagging sites. For the Type III‐A/B system, the His tag‐encoding sequence was inserted before the stop codon. For the Type I‐B and I‐C systems, the His tag‐encoding sequence was inserted behind the TTC due to the limitation of the PAM sequence. (B) Transformants were randomly selected and verified by PCR using the primer pair csm10His‐exm‐F and 10His‐R (depicted in panel A). The HB27 genomic DNA was used as the negative control (ck). (C) PCR products were subjected to DNA sequencing. (D) Western blot detection of the Csm3 protein carrying the His tag. M, marker. A purified 26 kDa recombinant protein carrying the His tag was used as the positive control (+). PAM, protospacer‐adjacent motif.

In situ gene tagging is important for resolving gene function under physiological conditions. In this study, we employed the CRISPR‐based genome editing strategy to achieve a His tag insertion in the *csm3* gene. Since the type III system is not restricted by PAM sequences, the crRNA encoded by the mini‐CRISPR on the editing plasmid targeted the sequence spanning the stop codon TAG of the *csm3* gene. In the donor sequence, the tag‐encoding 30‐bp sequence was inserted before the stop codon (Figure 7A). Since the type I system is restricted by the PAM sequence, we inserted the 30‐bp sequence between the PAM sequence and target sequence to change the relative position of the PAM and the target sequence so that the type I system could no longer recognize and cleave the target of mutants (Figure 7A). We performed PCR validation on the transformants using the specific primer 10His‐R. The results showed that no band was amplified from the wild‐type strain, and thus, the wild‐type strain was selected as the negative control. The insertion efficiency of 10 × His tag (30‐bp sequence) was 87.5% for type III‐A/B system (14/16), 93.8% for type I‐C system (15/16), and 100% for type I‐B system efficiency (16/16) (Figure 7B). DNA sequencing results also showed that the 10 × His tag was precisely inserted into the mutants at the target position as verified by PCR (Figure 7C). Csm3 is the backbone protein of the effector complex of the type III‐A system, and inserting a 10 × His tag in front of its stop codon will facilitate direct purification of this complex in *T. thermophilus* HB27 in vivo, thus alleviating the problem of heterologous purification in *Escherichia coli*. In addition, Western blots detected the expression of the Csm3 protein carrying a 10 × His tag (Figure 7D). The above results indicated that in situ tagging could be easily achieved using this CRISPR‐based genome editing strategy.

### Construction of the reporter gene system for *T. thermophilus* HB27

**Figure 8:**
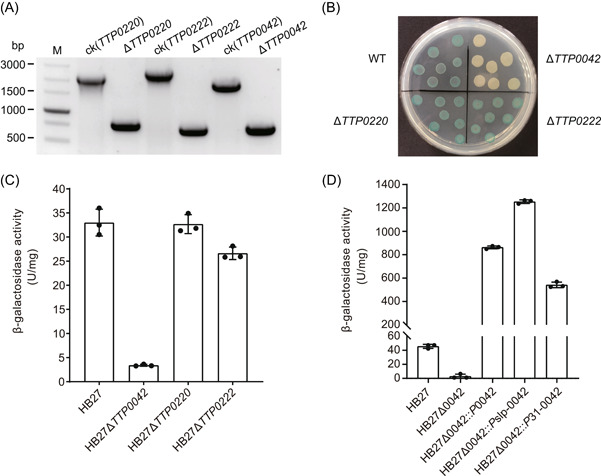
Construction of the reporter gene system for *Thermus thermophilus* HB27. (A) PCR verification of the β‐galactosidase gene locus in the wild‐type and knockout strains. (B) Phenotype of the wild‐type HB27 strain and 3 β‐galactosidase‐knockout strains on the X‐Gal plate. (C) β‐galactosidase activity of the wild‐type HB27 strain and 3 β‐galactosidase‐knockout strains. (D) Comparison of promoter activities based on the reporter gene system. M, DNA marker. Error bars indicate the standard deviation of three independent replicates.

An efficient reporter gene system is an important tool for studying gene expression regulation. In *T. thermophilus*, the heat‐stable β‐galactosidase gene can be used as a reporter gene, but the genome of *T. thermophilus* HB27 encodes multiple β‐galactosidase genes with background activities, which may interfere with the determination of reporter gene expression levels. There are two β‐galactosidase genes encoded on the HB27 genome, *TTP0042* and *TTP0220*, and *TTP0222* is on the megaplasmid pTT27^62^. We knocked out them using our CRISPR‐based genome editing strategy to eliminate their native enzyme activity. Based on the type III‐A/B system, we constructed three editing plasmids for knocking out these three β‐galactosidase genes and successfully obtained pure mutant strains with these three genes deleted (Figure 8A). Editing plasmids were cured by screening on antibiotic‐free plates. To determine whether each knockout strain still had β‐galactosidase activity, we added the knockout strains dropwise to TB plates containing 100 µg/ml X‐Gal with the wild‐type strain as the control. It was observed that on the same plate, the Δ*TTP0220* strain and Δ*TTP0222* strain still metabolized X‐Gal to generate a blue product and that these two deletion strains exhibited no difference from the wild‐type strain. However, the Δ*TTP0042* strain was still yellow on the X‐Gal plate, indicating that it could not metabolize X‐Gal and lacked β‐galactosidase activity (Figure 8B). We also compared the β‐galactosidase activity of the wild type and that of the three knockout strains by an ONPG (*o*‐nitrophenyl β‐d‐galactopyranoside) method^63^, ^64^. Similar to the X‐Gal plate assay results, we found that the β‐galactosidase activity of the Δ*TTP0042* strain was almost undetectable, while that of the Δ*TTP0220* and Δ*TTP0222* strains was consistent with the wild‐type strain (Figure 8C). These results showed that the knockout of the *TTP0042* gene completely removed the background β‐galactosidase activity of HB27, suggesting that the Δ*TTP0042* strain could be used as the host strain of the reporter gene system. To verify the feasibility of the reporter gene system, we designed three expression plasmids to compare the intensity of different promoters by this system. We used the promoter of the *TTP0042* gene, the *Pslp* promoter, and the *P31* promoter to express β‐galactosidase. After the transformation of these three expression plasmids into the Δ*TTP0042* strain, β‐galactosidase activities were detected. The results showed that the enzyme activity level driven by these promoters was more than 10 times as much as that of the wild‐type strain, and the enzyme activity expressed by the gene with the *Pslp* promoter reached a maximum of 1253 U/mg of total protein (Figure 8D), which was consistent with the previous reports on its strong promoter characteristics^65^, ^66^.

### Increase in SOD production by integrating the SOD‐expressing cassette into the genome

**Figure 9:**
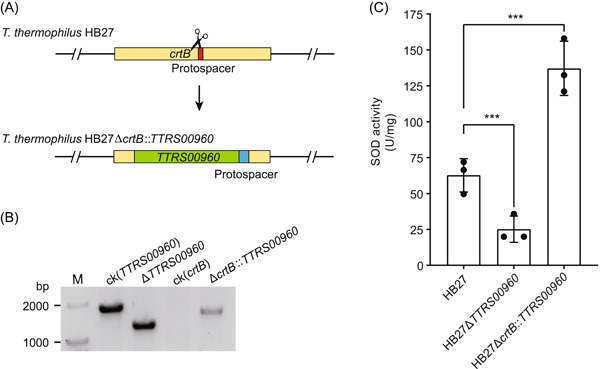
Integration of the SOD expression cassette into the genome to increase SOD production. (A) Schematic of integration of the SOD expressing cassette. (B) PCR verification of the *TTRS00960* or *crtB* gene locus in the wild‐type, Δ*TTRS00960* and Δ*crtB*::*TTRS00960* strains. ck, control group; M, DNA marker. The wild‐type strain was used as the control. (C) Comparison of SOD activities among wild‐type, Δ*TTRS00960*, and Δ*crtB*::*TTRS00960* strains. Error bars indicate the standard deviation of three independent replicates. ***p < 0.001. SOD, superoxide dismutase.

Thermophiles have great potential for industrial applications, and they are attractive chassis species for synthetic biology and the bioindustry. The enzyme technology has been widely used in research, development, and industrial manufacture. Reactive oxygen species (ROS) are important mediators for a variety of cellular processes, which act as second messengers in intracellular signaling^67^. Low levels of ROS are important for carrying out these cellular processes, but aberrant production of ROS often results in numerous diseases, including various neurological disorders^67^. Antioxidant enzymes can relieve the toxicity of ROS, and they attract increasing attention in terms of research and production^68^, ^69^. SOD is the primary ROS detoxifying enzyme of the cell that catalyzes the dismutation of superoxide radicals to hydrogen peroxide and molecular oxygen^70^, and has been widely used in medical, cosmetic, chemical, and other fields^71^. *T. thermophilus* HB27‐derived manganese SOD (Mn‐SOD) has extreme thermal stability and strong antioxidant capacity with promising applications^68^, ^72^. In this study, we utilized the genetic manipulation tool to modify *T. thermophilus* HB27 to improve its SOD production. We constructed a SOD gene (*TTRS00960*) integration plasmid based on the endogenous type I‐C CRISPR‐Cas system (Figure 9A,B). A constitutive promoter *P31*‐driven SOD expression cassette was successfully integrated into the locus of the nonessential gene *crtB*, thus increasing the SOD expression cassette number (Figure 9B and Figure S6B). We also constructed a SOD knockout strain (Δ*TTRS00960*) as a control (Figure 9B and Figure S6A). To verify the effectiveness of this integration, we measured the SOD level of this Δ*crtB::TTRS00960* strain. As we expected, the SOD enzyme activity level of the Δ*TTRS00960* strain was almost undetectable, whereas the SOD enzyme activity level of the Δ*crtB::TTRS00960* strain was significantly increased by more than 100% compared with that of the wild‐type strain (Figure 9C), indicating that our integration of the SOD expression cassette into *T. thermophilus* HB27 was effective. Our integration will provide a new perspective for industrial improvement and application of *T. thermophilus*.

## DISCUSSION

The study of thermophiles can elucidate the molecular mechanisms of life under high‐temperature conditions and reveal the biology of primitive life on Earth. Besides, thermophiles are also an important source of heat‐stable enzymes with great bioindustrial application potential. *T. thermophilus* is a model organism for the study of thermophiles. Efficient genetic manipulation tools are necessary to advance these studies. Currently, genetic manipulation tools based on the CRISPR‐Cas system have been widely used^23^, ^24^, ^25^, ^26^. Conventional genome editing strategies using exogenous CRISPR‐Cas systems can be limited by the complex intracellular environment and growth conditions of thermophiles.

*T. thermophilus* HB27 genome encodes multiple CRISPR‐Cas systems. Considering this, we developed genome editing tools based on endogenous CRISPR‐Cas systems of *T. thermophilus* HB27. First, we systematically examined the in vivo nucleic acid interference activity of the endogenous type I‐B, I‐C, and III‐A/B CRISPR‐Cas systems. We found that different types of endogenous CRISPR–Cas systems of *T. thermophilus* HB27 actively interfered with the self‐targeting plasmids, thus reducing CFUs by more than three orders of magnitude (Figure 2B). Using the plasmid that carried a self‐targeting mini‐CRISPR sequence and a donor, genetic manipulations such as gene knockout, site‐specific mutagenesis, and in situ tagging were highly efficient in *T. thermophilus* HB27. Further, we systematically compared the genome editing efficiency of the different types of endogenous CRISPR‐Cas systems (including Type I‐B, I‐C, IIIA/B). Overall, the Type I system exhibited more efficient gene editing than the Type III system, which might be because the Type I and Type III specifically targeted DNA and RNA, respectively, which led to fewer DNA breaks mediated by the Type III system. Fewer DNA breaks were confirmed by the fact that the transformation efficiency of type III‐editing plasmids was much higher than that of type I‐editing plasmids. It should be noted that although we did not systematically identify the preference of the endogenous type I system for the PAM sequence, our selected TTC PAM displayed high efficiency in genetic manipulation. Whether the identification of PAM sequence preference can improve editing efficiency remains to be further investigated. Although type III system‐mediated gene editing was less efficient than the type I system, we easily obtained the desired mutants based on the type III system. More importantly, compared to the type I system, the type III system does not have a strict PAM sequence preference, which enables us to introduce mutations at almost any locus. Although genome editing of *T. thermophilus* HB27 using thermostable exogenous Cas9 (CaldoCas9) protein has been reported recently^47^, there is no doubt that the native CRISPR‐based genome editing tool we developed here enables more convenient genome editing of *T. thermophilus* HB27. Our strategy only requires a mini‐CRISPR expressing the corresponding crRNA and a donor DNA for repair without introducing additional Cas proteins, which can simplify the plasmid construction process and obtain high transformation efficiency and editing efficiency. In addition, multiple endogenous CRISPR systems have broadened the limitations of the PAM sequence compared with Cas9, allowing for a more flexible design of gene‐editing sites.

It is worth noting that although *T. thermophilus* HB27 is a polyploid strain, we can easily obtain the desired pure mutants by our genetic manipulation methods. At present, genetic manipulation of polyploid strains based on endogenous CRISPR‐Cas systems has only been successful in *H. hispanica* ^30^. Therefore, the genome editing technique developed based on the endogenous CRISPR‐Cas system in this study will provide a reference for genetic manipulation of polyploid strains. In addition, we constructed a reporter gene system for *T. thermophilus* using this genetic manipulation tool and validated the ability of this system to assess promoter activity, which will facilitate the study of transcription factor function, protein expression, and other aspects of *T. thermophilus*. Since thermophiles have great potential for industrial applications, we applied our developed gene editing tool to modify *T. thermophilus* HB27. The SOD derived from *T. thermophilus* HB27 is highly resistant to high temperatures and exhibits high antioxidant capacity^68^, ^72^. In this study, we successfully achieved a significant increase in SOD production by integrating an additional SOD expression cassette into the genome of *T. thermophilus* HB27 (Figure 9C), which will provide the reference for the industrial modification and application of *T. thermophilus* HB27. Many prokaryotic species possess endogenous CRISPR‐Cas systems, particularly types I and III^18^, ^73^. With the development of genome sequencing technology and bioinformatics, native CRISPR‐Cas systems can be rapidly identified from specific genomes. Our study and the other works^29^, ^30^, ^31^, ^35^ provide a framework for genome editing based on endogenous CRISPR‐Cas systems, which will facilitate the development of the endogenous CRISPR‐Cas system into a next‐generation genome editing technology in prokaryotes.

In summary, our results show that all the endogenous type I‐B, I‐C, and III‐A/B CRISPR‐Cas systems of *T. thermophilus* can be employed for genome editing including gene knockout, site‐specific mutagenesis, and in situ tagging. Using our developed genetic manipulation tool, we constructed a reporter gene system for *T. thermophilus* and significantly increased its SOD production by genome modification. Our findings will provide a reference for genome editing of other prokaryotes based on the endogenous CRISPR‐Cas system, especially for polyploid species and extreme microorganisms. This study will facilitate genetic manipulations and the resolution of gene function of *T. thermophilus* and allow *T. thermophilus* to become an attractive chassis species used for synthetic biology and the bioindustry.

## MATERIALS AND METHODS

### Strains, culture conditions, and transformation

The *T. thermophilus* HB27 and derivatives constructed in this study are listed in Table S1. *T. thermophilus* HB27 and the derived stains were cultured in the modified TB medium^74^ (each liter of medium contained 8 g of tryptone, 4 g of yeast extract, 3 g of NaCl, 367 mg of NaHCO_3_, 31.34 mg of MgSO_4_, 0.95 mg of KCI, 36.75 mg of CaCl_2_ **·**2H_2_O, and 150.44 mg of MgCl_2_ **·**6H_2_O; the pH was adjusted to 7.5 with NaOH) at 65°C with 200 rpm shaking. Transformation of the *T. thermophilus* was conducted as a natural transformation method^75^ with some modifications. The overnight culture was inoculated into fresh TB medium at 4% and incubated with shaking at 65°C for 2.5 h. The culture (1 ml) was mixed with plasmids, incubated with shaking at 65°C for 2.5 h, and then spread on plates. For the selection of the *T. thermophilus* transformants, the medium had 20 μg/ml kanamycin.

The *E. coli* DH5α was used as the host strain for plasmid construction and cultured in the Luria–Bertani medium (10 g/l tryptone, 5 g/l yeast extract, 10 g/l NaCl) at 37°C with 180 rpm shaking^76^. For *E. coli*, 30 μg/ml kanamycin was added to the medium when needed.

### Genomic DNA extraction, sequencing, and annotation

Overnight *T. thermophilus* HB27 culture was inoculated into the fresh TB medium at 2%, and 5 ml of cell culture was harvested by centrifugation when the culture grew to OD600 ~1.0. The resulting cell pellet was used to extract genomic DNA. Total DNA extraction was conducted according to the instructions of the bacterial genomic DNA extraction kit (Tiangen, DP302). About 3 µg of genomic DNA was used for sequencing on Illumina MiSeq and Oxford Nanopore platforms, 614,258 of 2 × 150 bp pair‐end reads were obtained by Illumina sequencing, and 60,736 reads were obtained by Oxford Nanopore. Genome sequence assembly was performed using Unicycler (0.4.9)^77^. The coding functions of the obtained sequences were predicted using Prokka (1.12)^78^. Further annotations of the gene function were conducted by comparing the predicted gene sequences against the Refseq and nr database using BLAST+ (2.5.0+)^79^. The CRISPR arrays on the assembled genome were predicted using MinCED (0.4.2).

### Plasmid construction

Plasmids used or constructed in this study are listed in Table S1, and the primers used for plasmid construction or PCR validation are listed in Tables S2 and S3. PCR amplification was performed using the Phanta Max Super‐Fidelity DNA polymerase (Vazyme, P505). DNA fragments were digested and ligated using the restriction enzymes and T4 DNA ligase purchased from Thermo Fisher Scientific.

To construct the self‐targeting plasmid, spacers that matched with the kanamycin resistance gene were cloned between two *Bbs* I restriction sites on the pRKS‐AC shuttle vector. Spacer fragments were generated by annealing two complementary single‐stranded DNAs. The two homologous arms of the donor DNA were separately amplified using the HB27 genome as the template and connected using splicing by overhang extension PCR (SOE PCR). Plasmids for genome editing were constructed in two steps. First, spacers that matched the editing site were cloned between two *Bbs* I restriction sites on the pRKP31‐AC shuttle vector. Then, the resulting plasmid was digested with *Sal* I and *Nhe* I, and the donor DNA was cloned between these two sites by the T5 exonuclease‐dependent assembly (TEDA)^80^. To construct plasmids that expressed β‐glycosidase, the *TTP0042* fragment amplified from the genomic DNA was cloned into pRKP31, which was predigested with *Sal* I and *Nhe* I. Then, the *P31* promoter was replaced with other promoters based on the pRKP31‐*TTP0042* by TEDA. Plasmids were validated by DNA sequencing before being transformed into the *T. thermophilus* HB27 cells.

### Mutant screening and validation

The empty plasmid, self‐targeting plasmids, and editing plasmids were transformed into *T. thermophilus* HB27 cells using the method described above. The colonies on the selective plates were counted after 48 h of incubation. For mutant screening, transformants were randomly picked (unless specified) and inoculated into the culture medium. After incubation for 4 h, the cells were then subjected to PCR validation using primers listed in Table S2. The resulting PCR products were analyzed by agarose gel electrophoresis and confirmed by DNA sequencing (Tsingke).

### Western blot analysis

The Csm3‐His‐tagged strain was cultured in the TB medium. When the OD_600_ of the culture reached 2.0, cells (20 ml) were collected by centrifugation. Phosphate buffer (50 mM) was used to resuspend the cell pellets. Afterward, the resulting bacterial suspension was lysed by sonication. Then, the mixture was centrifuged (13,000 rpm, 20°C, 30 min), and the supernatant was fractionated through 12% SDS‐PAGE. Fractionated proteins were transferred onto a PVDF membrane using the Semi‐Dry Electrophoretic Transfer Cell system (Bio‐Rad). Antibody incubation and visualization were performed as previously reported^29^.

### β‐glucosidase activity assay for *T. thermophilus*

The β‐galactosidase assay was performed using an ONPG method^63^, ^64^. The reporter plasmids were transformed into the *T. thermophilus* HB27Δ*TTP0042* strain. Three single colonies were randomly selected for each assay. Transformants carrying different reporter plasmids were grown in the TB medium. Then, cells grown to the log phase (OD_600_ ~1.0) were collected by centrifugation, and cell pellets were resuspended in 10 mM Tris‐HCl at pH 8.0. Next, crude cell lysates were obtained by ultrasonic lysis. Cell debris in the lysates was removed by centrifugation (13,000 rpm, 20°C, 30 min) before the ONPG assay. The protein content of the cellular extracts was determined by the Bradford Protein Assay Reagent (Tiangen, PA102). For each assay, 50 μl of the supernatant was added to 450 μl of reaction buffer, which contained 2.8 mM ONPG and 50 mM sodium phosphate at pH 6.5. Samples were incubated at 65°C for 20 min, and the reaction was stopped by adding an equal volume of 1 M sodium carbonate. The absorbance of *o*‐nitrophenol was determined by using a microplate reader (Victor Nivo 3S; PerkinElmer) at 420 nm. One unit of specific enzyme activity was defined as 1 nmol ρ‐nitrophenol produced per min per mg total protein.

### SOD activity assay for *T. thermophilus*

Strains for the SOD activity assay were cultured in the TB medium. When the OD _600_ of the culture reached 0.8, cells (1 ml) were collected by centrifugation. The cell pellets were resuspended in 50 mM phosphate buffer and sonicated. The cell debris was removed by centrifugation (13,000 rpm, 20°C, 30 min), and the supernatant was used for the SOD activity assay. The SOD activity assay was performed based on a WST‐1 (water‐soluble tetrazolium) method^81^. The SOD level of the cellular extract was determined according to the instruction of the Total Superoxide Dismutase Assay Kit with WST‐1 (Biosharp, BL902A). The protein content of the cellular extracts was determined as described above. One SOD activity unit was defined as the level of SOD per mg of total protein.

### Statistical analysis

All of the histogram drawings and statistical analysis were completed by the software GraphPad Prism 8 (GraphPad Software Inc.). Data are expressed as the mean ± standard deviation (SD). Between‐group difference was compared using the unpaired two‐sample *t*‐test. *p* < 0.05 was considered statistically significant.

## AUTHOR CONTRIBUTIONS

Yingjun Li, Haijuan Li, Jinting Wang, and Junwei Wei designed and conceived the project. Jinting Wang and Junwei Wei performed the experiments. Yingjun Li, Jinting Wang, and Junwei Wei analyzed the data. Junwei Wei and Jinting Wang drafted the manuscript under the guidance of Yingjun Li.

## ETHICS STATEMENT

This study has no animal or human experiments. There are no ethical issues involved.

## CONFLICT OF INTERESTS

The authors declare no conflict of interests.

## DATA AVAILABILITY

All data generated or analyzed during this study are included in this article and its supporting information files.

## Supporting information

Supporting information.

Supporting information.
