# Correction to “High‐efficiency genome editing of an extreme thermophile *Thermus thermophilus* using endogenous type I and type III CRISPR‐Cas systems”

**DOI:** 10.1002/mlf2.70010

**Published:** 2025-04-04

Wang J, Wei J, Li H, Li Y. High‐efficiency genome editing of an extreme thermophile *Thermus thermophilus* using endogenous type I and type III CRISPR‐Cas systems. mLife. 2022;1:412–427. https://doi.org/10.1002/mlf2.12045

In paragraph 4 of the “Introduction” section, the text “*Thermus thermophilus* is an extremely thermophilic Gram‐positive bacterium” was incorrect. This should have read: “*Thermus thermophilus* is an extremely thermophilic Gram‐negative bacterium.”

In Table 1, the text “GTTGCAAGGGATTGARCCCCGTAAGGGGATTGCGAC” was incorrect. This should have read: “GTTGCAAGGGATTGAGCCCCGTAAGGGGATTGCGAC”.

In Figure 2, panel B contained inaccurate data due to a miscalculation in the statistical analysis. The corrected version of this figure is provided below.

**Figure 2:**
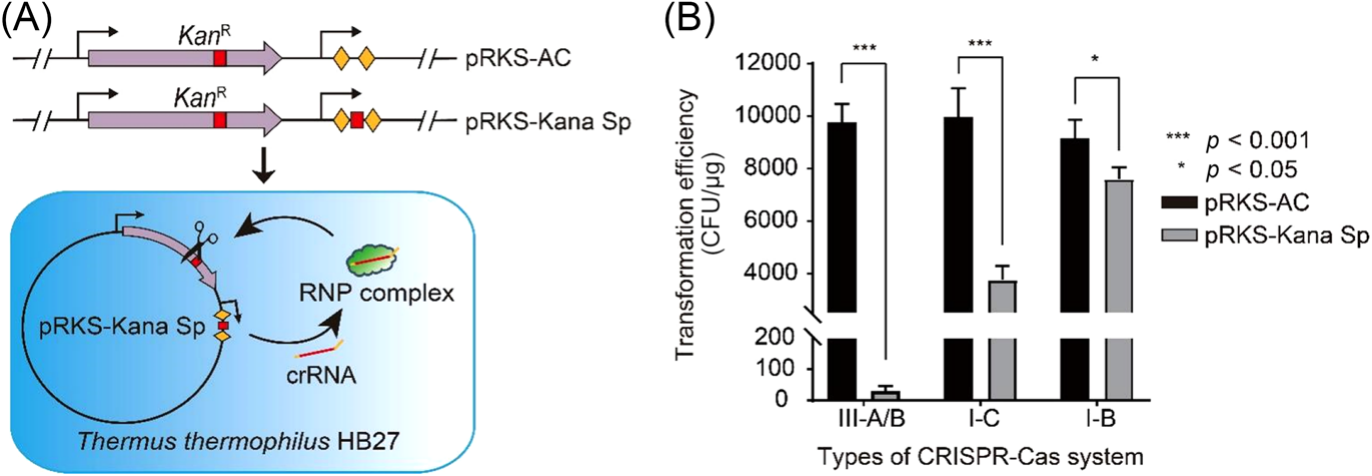
.

In paragraph 2 of “Functional identification of different types of endogenous CRISPR‐Cas systems” subsection of the “Results” section, the text “It was shown that the transformation efficiency of the self‐targeting plasmid pRKS‐Kana Sp was significantly lower than that of the control plasmid pRKS‐AC and that transformation efficiency mediated by I‐B, I‐C, and III‐A/B systems was reduced by over 99.5% (Figure 2B).” was incorrect. This should have read: “It was shown that the transformation efficiency of the self‐targeting plasmid pRKS‐Kana Sp was significantly lower than that of the control plasmid pRKS‐AC, particularly with the transformation efficiency mediated by III‐A/B systems reduced by over 99.5% (Figure 2B).”

In paragraph 1 of “Gene knockout based on different types of endogenous CRISPR‐Cas systems” subsection of the “Results” section, the text “As mentioned above, we have verified that the endogenous CRISPR‐Cas systems of *T. thermophilus* HB27 could actively interfere with self‐targeting plasmids to reduce colony forming units (CFUs) by three orders of magnitude (Figure 2B).” was incorrect. This should have read: “As mentioned above, we have verified that the endogenous CRISPR‐Cas systems of *T. thermophilus* HB27 could actively interfere with exogenous plasmids to reduce the number of colony‐forming units (CFUs) (Figure 2B).”

In paragraph 2 of the “Discussion” section, the text “We found that different types of endogenous CRISPR‐Cas systems of *T. thermophilus* HB27 actively interfered with the self‐targeting plasmids, thus reducing CFUs by more than three orders of magnitude (Figure 2B).” was incorrect. This should have read: “We found that different types of CRISPR‐Cas systems in *T. thermophilus* HB27 actively interfered with the exogenous self‐targeting plasmids (Figure 2B).”

We apologize for these errors.

