# Supplementary material for: High‐efficiency genome editing of an extreme thermophile Thermus thermophilus using endogenous type I and type III CRISPR‐Cas systems
Source: mLife. 2022 Dec 7;1(4):412–27. doi: 10.1002/mlf2.12045 (PMC10989782; doi:10.1002/mlf2.12045)

**Table S1.** Strains and plasmids used in this study.

| **Strains/plasmid** | **Description** | **Source/reference** |
| --- | --- | --- |
| **Strains** | | |
| *E. coli* DH5α | host for plasmid construction | TsingKe Biotech |
| *T. thermophilus* HB27 | wild-type *T. thermophilus* HB27 strain | Lab storage |
| Δ*crtB* | HB27 with *crtB* gene deleted | This study |
| Δ*cas3* | HB27 with *cas3* gene deleted | This study |
| *csm3*_(D34Q)_ | HB27 with point mutation at the 34^th^ amino acid of *csm3* gene locus | This study |
| *csm3*_(F92L)_ | HB27 with point mutation at the 92^nd^ amino acid of *csm3* gene locus | This study |
| *csm3* His-tagged01 | HB27 with his tagged *csm*3 gene in front of its stop codon. | This study |
| *csm3* His-tagged02 | HB27 with his tagged *csm*3 gene behind its TTC PAM sequence. | This study |
| Δ*TTP0220* | HB27 with *TTP0220* gene deleted | This study |
| Δ*TTP0222* | HB27 with *TTP0222* gene deleted | This study |
| Δ*TTP0042* | HB27 with *TTP0042* gene deleted | This study |
| Δ*TTRS00960* | HB27 with *TTRS00960* gene deleted | This study |
| Δ*crtB*::*TTRS00960* | HB27 with integrated with SOD expression cassette at the *crtB* gene locus | This study |
|  |  |  |
| **Plasmids** | | |
| pRKS | *E. coli - T. thermophilus* shuttle vector with P*slp* promoter | This study |
| pRKP31 | *E. coli - T. thermophilus* shuttle vector with P31 promoter | This study |
| pRKS-AC1 | pRKS carrying mini-CRISPR array for type III CRISPR-Cas system | This study |
| pRKS-AC2 | pRKS carrying mini-CRISPR array for type I-C CRISPR-Cas system | This study |
| pRKS-AC3 | pRKS carrying mini-CRISPR array for type I-B CRISPR-Cas system | This study |
| pRKS-Kana-Sp1 | pRKS-AC1 carrying a kanamycin resistant gene-targeting spacer | This study |
| pRKS-Kana-Sp2 | pRKS-AC2 carrying a kanamycin resistant gene-targeting spacer | This study |
| pRKS-Kana-Sp3 | pRKS-AC3 carrying a kanamycin resistant gene-targeting spacer | This study |
| pRKP31-crtB-Sp1-LR | Editing plasmid for *crtB* gene deletion based on type III CRISPR-Cas system | This study |
| pRKP31-crtB-Sp2-LR | Editing plasmid for *crtB* gene deletion based on type I-C CRISPR-Cas system | This study |
| pRKP31-crtB-Sp3-LR | Editing plasmid for *crtB* gene deletion based on type I-B CRISPR-Cas system | This study |
| pRKS-ICcas3-Sp1-LR | Editing plasmid for *cas3* gene deletion based on type III CRISPR-Cas system | This study |
| pRKS-ICcas3-Sp2-LR | Editing plasmid for *cas3* gene deletion based on type I-C CRISPR-Cas system | This study |
| pRKS-ICcas3-Sp3-LR | Editing plasmid for *cas3* gene deletion based on type I-B CRISPR-Cas system | This study |
| pRKP31-KOcrtB-Sp1_  1-7 | Editing plasmid with different length donor for *crtB* gene deletion based on type III CRISPR-Cas system | This study |
| pRKP31-KOcrtB-Sp2_  1-7 | Editing plasmid with different length donor for *crtB* gene deletion based on type I-C CRISPR-Cas system | This study |
| pRKP31-KOcrtB-Sp3_  1-7 | Editing plasmid with different length donor for *crtB* gene deletion based on type I-B CRISPR-Cas system | This study |
| pRKP31-csm3 D34Q | Editing plasmid for *csm3* gene point mutation based on type III CRISPR-Cas system | This study |
| pRKP31-csm3 F92L-Sp2 | Editing plasmid for *csm3* gene point mutation based on type I-C CRISPR-Cas system | This study |
| pRKP31-csm3 F92L-Sp3 | Editing plasmid for *csm3* gene point mutation based on type I-B CRISPR-Cas system | This study |
| pRKP31-csm3 10xHis-Sp1 | Editing plasmid for *csm3* *in situ* tagging based on type III CRISPR-Cas system | This study |
| pRKP31-csm3 10xHis-Sp2 | Editing plasmid for *csm3* *in situ* tagging based on type I-C CRISPR-Cas system | This study |
| pRKP31-csm3 10xHis-Sp3 | Editing plasmid for *csm3* *in situ* tagging based on type I-B CRISPR-Cas system | This study |
| pRKP31-TTP0220 Sp1- LR | Editing plasmid for *TTP0220* gene deletion based on type III CRISPR-Cas system | This study |
| pRKP31-TTP0222 Sp1-LR | Editing plasmid for *TTP0222* gene deletion based on type III CRISPR-Cas system | This study |
| pRKP31-TTP0042 Sp1-LR | Editing plasmid for *TTP0042* gene deletion based on type III CRISPR-Cas system | This study |
| pRKS-TTP0420 | *TTP0042* expression plasmid with P*slp* promoter | This study |
| pRKP31-TTP0420 | *TTP0042* expression plasmid with P31 promoter | This study |
| pRKP*_0042_*-TTP0420 | *TTP0042* expression plasmid with P*_0042_* promoter | This study |
| pRKP31-TTRS00960 KO- Sp2 | Editing plasmid for *TTRS00960* gene deletion based on type I-C CRISPR-Cas system | This study |
| pRKP31-TTRS00960 integration- Sp2 | Editing plasmid for *TTRS00960* gene integration based on type I-C CRISPR-Cas system | This study |

**Table S2. Primes used in this study.**

| **Primer** | **Sequence (5****′→3′)** | |
| --- | --- | --- |
| **For construction of self-targeting and editing plasmids** | | |
| Kana-Sp1-F | | CGACCACTTTCTCTAAGTATCCACCTGAATCATAAATCGGCAAA |
| Kana-Sp1-R | | CAACTTTGCCGATTTATGATTCAGGTGGATACTTAGAGAAAGTG |
| Kana-Sp2/3-F | | AAACGTGTGCAAGGACCGACAACATTTCTACCATCCTTGAC |
| Kana-Sp2/3-R | | CAACGTCAAGGATGGTAGAAATGTTGTCGGTCCTTGCACAC |
| crtB-Sp1-F | | CGACGCTCCGCCTCCGTCCCGAGCCGCACGGGGCCGAGGTCGGT |
| crtB-Sp1-R | | CAACACCGACCTCGGCCCCGTGCGGCTCGGGACGGAGGCGGAGC |
| crtB-Sp2/3-F | | AAACCTGACCGACCTCGGCCCCGTGCGGCTCGGGACGGAGGC |
| crtB-Sp2/3-R | | CAACGCCTCCGTCCCGAGCCGCACGGGGCCGAGGTCGGTCAG |
| crtB-L-R | | AGGCCTTGAGGACGCGGAGGAGGGCTTTCCAGTC |
| crtB-R-F | | CCTCCGCGTCCTCAAGGCCTGGGAACGGGCCCTC |
| crtB-100D-1L-F | | ggggattgcgaccgcgtcgaACATCTCTGCGGAGTGTAGC |
| crtB-100D-2/3L-F | | gaggattgaaaccgcgtcgaACATCTCTGCGGAGTGTAGC |
| crtB-100D-R-R | | GTGATGGTGATGGTGgctagACGAGAAGGGGGCCCATCAGGG |
| crtB-200D-1L-F | | ggggattgcgaccgcgtcgaGGAAAGGCCGGTCATGGCTT |
| crtB-200D-2/3L-F | | gaggattgaaaccgcgtcgaGGAAAGGCCGGTCATGGCTT |
| crtB-200D-R-R | | GTGATGGTGATGGTGgctagGTTGTTGGGGTCCAGGACCAC |
| crtB-300D-1L-F | | ggggattgcgaccgcgtcgaGGGCCTCCACGTCTTCCGC |
| crtB-300D-2/3L-F | | gaggattgaaaccgcgtcgaGGGCCTCCACGTCTTCCGC |
| crtB-300D-R-R | | GTGATGGTGATGGTGgctagCCTCCAGGACCCAAAGGGCT |
| crtB-400D-1L-F | | ggggattgcgaccgcgtcgaCCGGTCCCCAGGTCCTCGG |
| crtB-400D-2/3L-F | | gaggattgaaaccgcgtcgaCCGGTCCCCAGGTCCTCGG |
| crtB-400D-R-R | | GTGATGGTGATGGTGgctagGCGTCCCGGTAGCGGCCGTAG |
| crtB-500D-1L-F | | ggggattgcgaccgcgtcgaGAGGTGCTCCAGGACGCCCT |
| crtB-500D-2/3L-F | | gaggattgaaaccgcgtcgaGAGGTGCTCCAGGACGCCCT |
| crtB-500D-R-R | | GTGATGGTGATGGTGgctagGAAGGGGGTGTAGACCCGGT |
| crtB-600D-1L-F | | ggggattgcgaccgcgtcgaGGAGCCTTGCCCGGAGGAAGGT |
| crtB-600D-2/3L-F | | gaggattgaaaccgcgtcgaGGAGCCTTGCCCGGAGGAAGGT |
| crtB-600D-R-R | | GTGATGGTGATGGTGgctagGAAGCCCCGGGTCTTCCCG |
| crtB-700D-1L-F | | ggggattgcgaccgcgtcgaTAGGCGGCGAGCATGGCC |
| crtB-700D-2/3L-F | | gaggattgaaaccgcgtcgaTAGGCGGCGAGCATGGCC |
| crtB-700D-R-R | | GTGATGGTGATGGTGgctagCCGTCCAGCCGGTCCCG |
| ICcas3-Sp1-F | | cgacGTCGGTGGACCTTGCGCATCTTAGAGGGGCGGTTGGAGAA |
| ICcas3-Sp1-R | | caacTTCTCCAACCGCCCCTCTAAGATGCGCAAGGTCCACCGAC |
| ICcas3-Sp2/3-F | | aaacTCCAACCGCCCCTCTAAGATGCGCAAGGTCCACCGAC |
| ICcas3-Sp2/3-R | | caacGTCGGTGGACCTTGCGCATCTTAGAGGGGCGGTTGGA |
| ICcas3-1L-F | | gtaaggggattgcgaccgcgTATCGTAGCCCTCCTGATGGGCAG |
| ICcas3-2/3L-F | | cggtgaggattgaaaccgcgTATCGTAGCCCTCCTGATGGGCAG |
| ICcas3-L-R | | GCCAGACGTAGAGGAGTTTGCTGGCCGCCTCACCCCTCT |
| ICcas3-R-F | | GGCCAGCAAACTCCTCTACGTCTGGCGTGGGTCATAT |
| ICcas3-R-R | | GATGGTGATGGTGATGGTGGAGGTGGCGAGAAGTAAGCGGGAAACT |
| csm3_D34Q_-Sp1-F | | CGACTTGTCCAGGTCGCCGATGGCCATCTGGTCCCGGCTCATCC |
| csm3_D34Q_-Sp1-R | | CAACGGATGAGCCGGGACCAGATGGCCATCGGCGACCTGGACAA |
| csm3_F92L_-Sp2/3-F | | AAACGGCCTGGCCCCGGAGAACGACGAGAGGTCTTTGGCAGT |
| csm3_F92L_-Sp2/3-R | | CAACACTGCCAAAGACCTCTCGTCGTTCTCCGGGGCCAGGCC |
| csm3_D34Q_-1L-F | | GTAAGGGGATTGCGACCGCGTCTGGGTCCAGTGGGCGCTTT |
| csm3_D34Q_-1L-R | | CGGGGTTCTGCAGGTCGCCGATGGCCATCTGGTCCC |
| csm3_D34Q_-1R-F | | GACCTGCAGAACCCCGTGGTCCGCAACCCCCTCACG |
| csm3_D34Q_-R-R | | GTGATGGTGATGGTGGCTAGGGCTCCTGACGGAACCTCTCT |
| csm3_D34Q_-2L-F | | CGGTGAGGATTGAAACCGCGTCTGGGTCCAGTGGGCGCTTT |
| csm3_D34Q_-3L-F | | CGTAGAGGATTGAAACCGCGTCTGGGTCCAGTGGGCGCTTT |
| csm3_D34Q_-2/3L-R | | AGGCCTAGGATGCGGGCCACGGGGTCCTTGGGGTCGG |
| csm3_D34Q_-2/3R-F | | TGGCCCGCATCCTAGGCCTGGCCCCGGAGAACGACGAG |
| csm3_10his_-Sp1-F | | CGACCCTAAAGGACCACTTCCTCCACCTTGAGCCTTTCCTTTAG |
| csm3_10his_-Sp1-R | | CAACCTAAAGGAAAGGCTCAAGGTGGAGGAAGTGGTCCTTTAGG |
| csm3_10his_-Sp2/3-F | | AAACCTCCACCCCGAAAGGCTTACGGAGGACCAGGAGGGCTG |
| csm3_10his_-Sp2/3-R | | CAACCAGCCCTCCTGGTCCTCCGTAAGCCTTTCGGGGTGGAG |
| csm3_10his_-1L-F | | GTAAGGGGATTGCGACCGCGATGAAGCTCAAGAAGGTGATCC |
| csm3_10his_-1L-R | | ATGATGATGGTGATGGTGATGGTGGTGATGAAGGACCACTTCCTCCACCTT |
| csm3_10his_-1R-F | | CATCACCACCATCACCATCACCATCATCATTAGGGGGCCTGGATGCGGGCGAC |
| csm3_10his_-R-R | | GATGGTGATGGTGATGGTGGTGGCGTAGGCGTTCGGCTCCTT |
| csm3_10his_-2L-F | | CGGTGAGGATTGAAACCGCGATGAAGCTCAAGAAGGTGATCC |
| csm3_10his_-3L-F | | CGTAGAGGATTGAAACCGCGATGAAGCTCAAGAAGGTGATCC |
| csm3_10his_-2/3L-R | | ATGATGATGGTGATGGTGATGGTGGTGATGGAAGTAGACCTGGCCGTAGC |
| csm3_10his_-2/3R-F | | CATCACCACCATCACCATCACCATCATCATCTCCACCCCGAAAGGCTTACG |
| 0220-Sp1-F | | CGACCCCTGAGCCCCCCGTAGCTGTAGACCTCGTCCAGGACCCG |
| 0220-Sp1-R | | CAACCGGGTCCTGGACGAGGTCTACAGCTACGGGGGGCTCAGGG |
| 0222-Sp1-F | | CGACCCCTTTGCGCCATCTCCTCCGAGTAGCCCGCGAGCCACTG |
| 0222-Sp1-R | | CAACCAGTGGCTCGCGGGCTACTCGGAGGAGATGGCGCAAAGGG |
| 0042-Sp1-F | | CGACCGGCGAGGCCGTGGCCCAGGAGGAGGTGGTGGGCGGCGCG |
| 0042-Sp1-R | | CAACCGCGCCGCCCACCACCTCCTCCTGGGCCACGGCCTCGCCG |
| 0220-1L-F | | GTAAGGGGATTGCGACCGCGGCCCTCCTCCTCTTCCTGC |
| 0220-1L-R | | CTCGGGGGGCACCTTGGCCTCGCTTAGGGCGAAG |
| 0220-1R-F | | GCCCTAAGCGAGGCCAAGGTGCCCCCCGAGAGGGTC |
| 0220-1R-R | | GATGGTGATGGTGATGGTGGCTCCTGCCGCAGGAGCCTG |
| 0222-1L-F | | GTAAGGGGATTGCGACCGCGCCGCCCCCCTGGACCGGA |
| 0222-1L-R | | GTACTCCACGCTGAGTACCCCCTCCAGGCTCCAC |
| 0222-1R-F | | AGCCTGGAGGGGGTACTCAGCGTGGAGTACCTGGA |
| 0222-1R-R | | GATGGTGATGGTGATGGTGGTAGGGCGATGCTGGCCGT |
| 0042-1L-F | | GTAAGGGGATTGCGACCGCGCCTCGTGGGCAGCTACGG |
| 0042-1L-R | | GCCACCAGCGCCTACGTGGACTTCCCCAGCCAG |
| 0042-1R-F | | GCCACCAGCGCCTACGTGGACTTCCCCAGCCAG |
| 0042-1R-R | | GATGGTGATGGTGATGGTGGGTGGACCTGGAGTTCTGGC |
| KO0960-Sp3-F | | AAACACCCCCATCGTGGGCATTGACGTCTGGGAGCACGCCT |
| KO0960-Sp3-R | | CAACAGGCGTGCTCCCAGACGTCAATGCCCACGATGGGGGT |
| KO0960-L-F | | GAGGATTGAAACCGCGTCGACTTCCTTGGGAGAGGCGGTCTTGGT |
| KO0960-L-R | | GCCGAGGAGTTCTTCAAGAAGGCCTGAT |
| KO0960-R-F | | TTGAAGAACTCCTCGGCGTAGCCTAGGTCAGGAAGCTTGAACGGGT |
| KO0960-R-R | | GTGATGGTGATGGTGGCTAGTGGAGGCCCTCACCATGGTGGTGGT |
| crtB-0960-L-R | | CATAGCCTTATAGCCTGAGGACGCGGAGGAGGGCTTTCCAGTC |
| Int0960-L-F | | AGGCTATAAGGCTATGGGGATCAGG |
| Int0960-R | | GCGGCAAGGGGCTTTGTGAGGAAG |
| 0960-crtB-R-F | | CAAAGCCCCTTGCCGCTCAAGGCCTGGGAACGGGCCCTC |
|  | |  |
| **For PCR screening and DNA sequencing** | | |
| crtB-exm-F | | TTAAAGGCTCGGAAAGGACGGC |
| crtB-exm-R | | GTAGAGGAGGTGGTAGGAGAAGTC |
| ICcas3-exm-F | | CCCTCTAAGACGGCCTTCTCACCTATG |
| ICcas3-exm-R | | GGCTTGTCCGCTGCTGGCGCGCAT |
| csm3 exm-F | | TGGCCTACGCCCTAAGGCGCGT |
| csm3 exm-R1 | | GTCTGATCAGCGCTCCAGGTCCTCCGTAAGCCTTTGGCCTCCTTGGCGTCCTCCGT |
| csm10His-exm-F | | TCCTCAGGGCCAAGCTCTTCTACAAC |
| 10His-R | | ATGATGATGGTGATGGTGATGGTGGTGATG |
| exam-20KO-F | | GGCTGGCCCACCTCCTCATG |
| exm-20KO-R | | CCTTCCACCTCCCTACTCCGCGAG |
| exm-22KO-F | | CCTGCCCAAGGGCGGCTGGTAC |
| exm-22KO-R | | TGGGCATCCCCAGGCGATCCAGGTAGTAG |
| exm-42KO-F | | GTGATCCCCGGGATCCTCTTCCTGAT |
| exm-42KO-R | | GCCTTCGTCCCGGCCTTCTTTGAG |
| exm-SOD KO-F | | GGACCGGGCTTCGTCTACCTGGAGACG |
| exm-SOD KO-R | | TACGCGGGCATTGGGGAGATCACCATCC |
| Int0960-R | | GCGGCAAGGGGCTTTGTGAGGAAG |


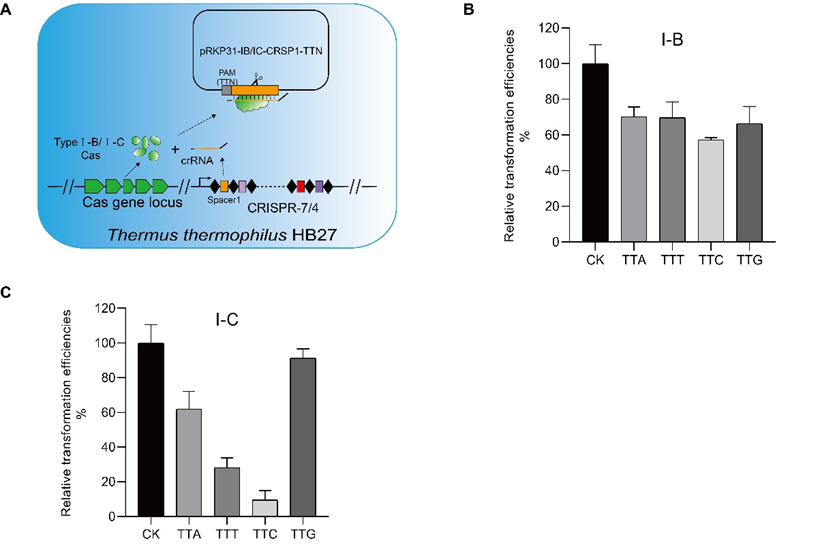
**Figure S1. Identification of interference activities and TTN PAM preferences for type I-B and I-C systems.** (A) Schematic diagram of *in vivo* interference activity validation with different target plasmids. Target plasmids (pRKP31-IB/IC-CRS1-TTN) carried a common protospacer with different PAM sequences (TTA, TTT, TTC, and TTG) adjacent to its 5’ end. The protospacer for type I-B and type I-C systems was designed based on the first spacer sequence of CRISPR-7 and CRISPR-4 respectively. (B) Relative transformation efficiency of target plasmids for type I-B system. (C) Relative transformation efficiency of target plasmids for type I-C system. Error bars indicate the standard deviation (SD) of three independent replicates. CK, plasmid without protospacer (pRKP31).

**Figure S2.** **The DNA sequencing results of gene knockout.** DNA sequencing peaks of PCR products of the wild-type (wt) and the *ctrB*-deleted (Δ*crtB*) strain (A), the *cas3*-deleted (Δ*cas3*) strain (B). Partial sequences of the *crtB*, *cas3* gene and the recombination arm are presented.


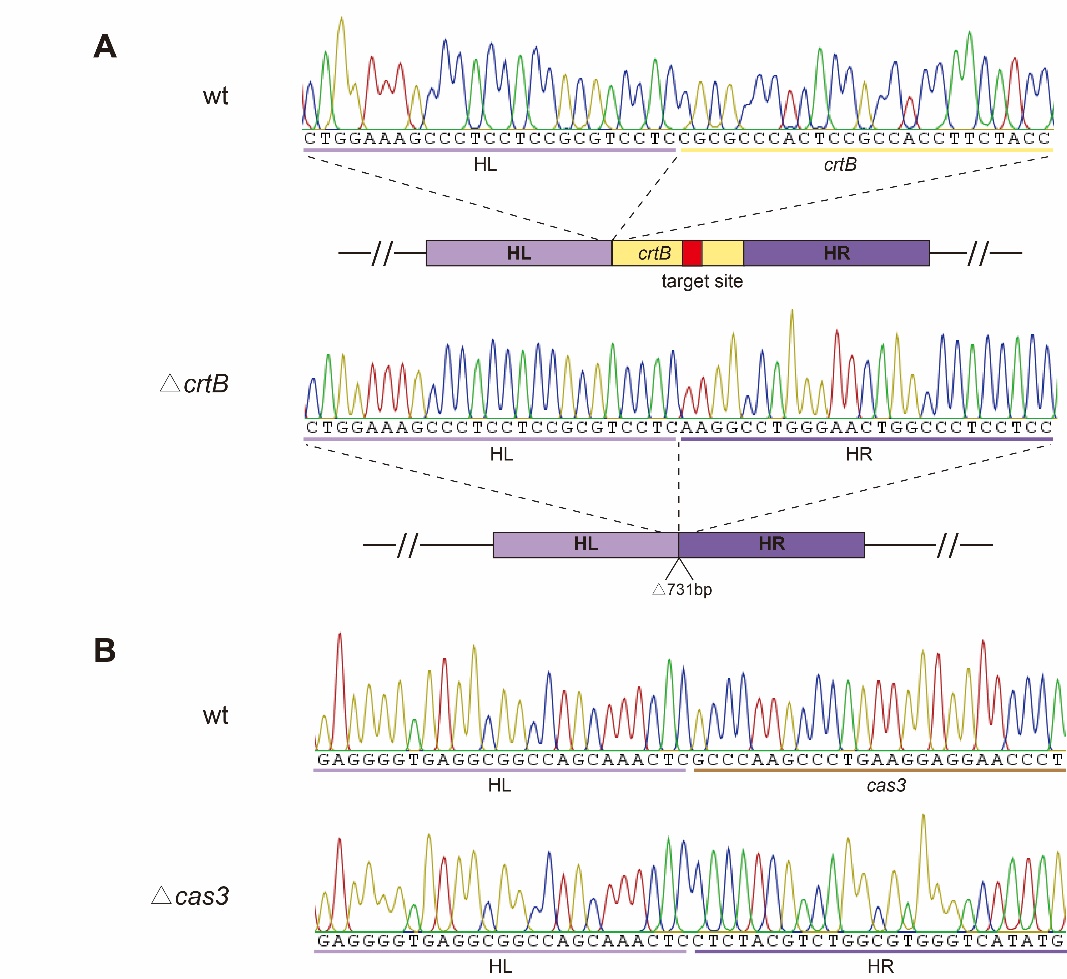


**Figure S3.** **Impact of donor size on transformation efficiency of editing plasmids.** (A) Effect of donor length on transformation efficiency of type III-A/B system-based plasmids. (B) Effect of donor length on transformation efficiency of type I-C system-based plasmids. (C) Effect of donor length on transformation efficiency of type I-B system-based plasmids. Error bars indicate the standard deviation (SD) of three independent replicates.


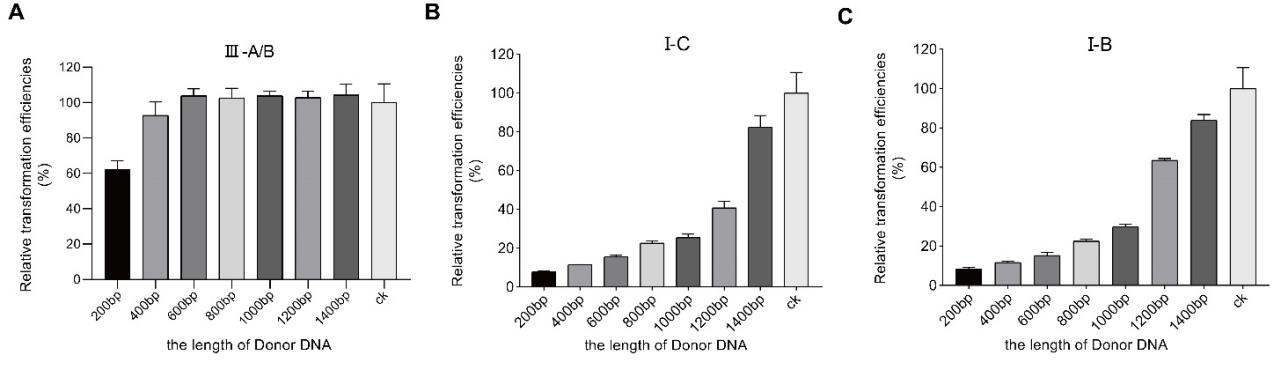


**Figure S4. Amplified *crtB* gene locus of the randomly selected white colonies.** For each plasmid with different length donors, 16 white colonies were randomly selected for PCR validation with primers crtB-exm-F/R. Plasmids were based on type III-A/B (A), I-C (B) and I-B (C) systems, respectively. M, DNA marker. ck, the wild-type strain was used as the control.


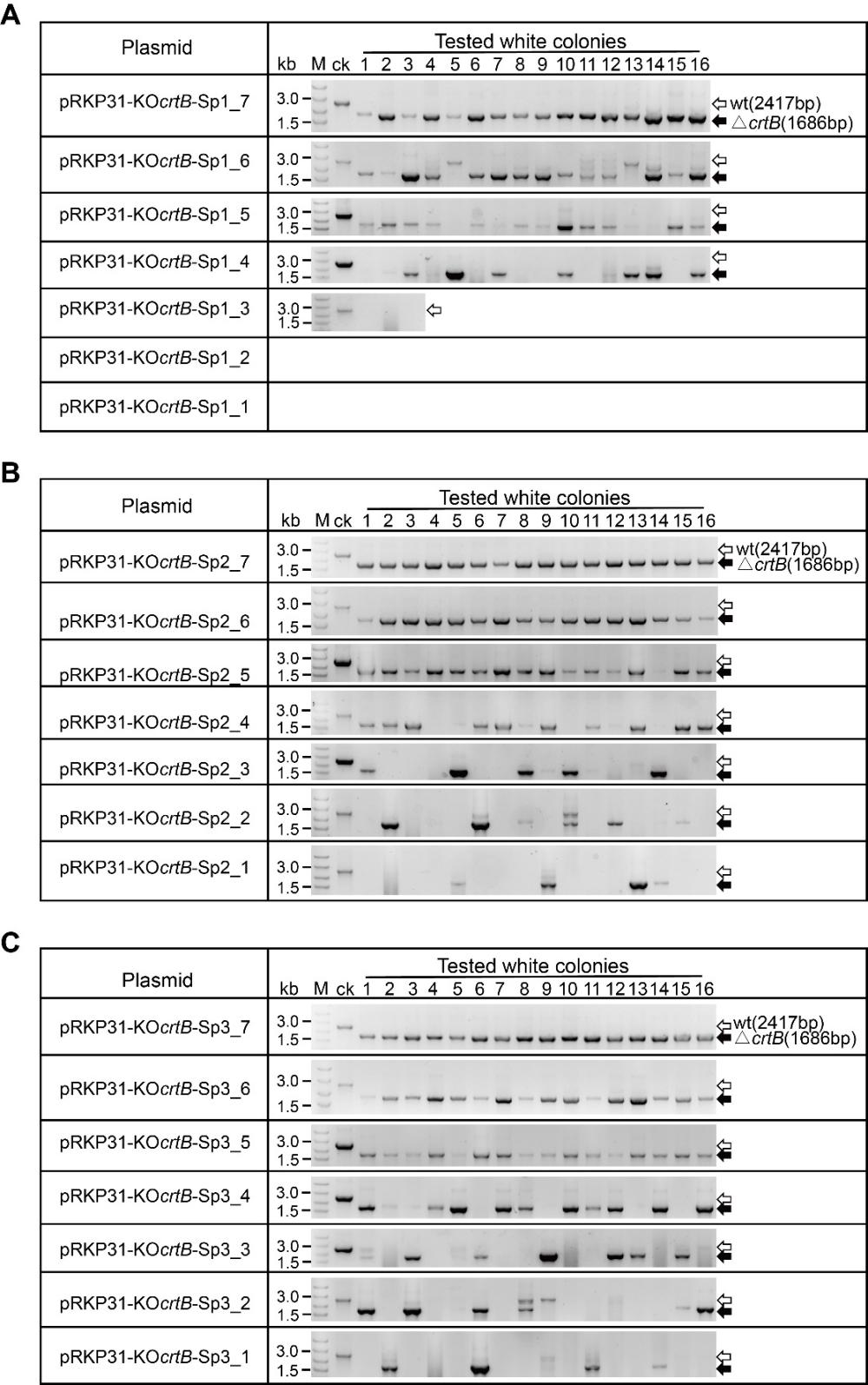


**Figure S5. The DNA sequencing results of gene point mutation.** Sequencing peaks of the PCR products amplified from the wild-type and point-mutated *csm3* gene loci. (A) Point-mutated *csm3* based on type III-A/B systems (G to C and C to G conversions). (B) Point-mutated *csm3* based on type I-B and I-C systems (T to C and C to A conversions). (C) Point-mutated *csm3* based on type III-A/B systems (G to A conversion). (D) Point-mutated *csm3* based on type I-B and I-C systems (T to A conversion). The red arrow marks the mutation site. The editing efficiency is also shown in the lower left of the figures (e.g. 8/16, 14/16).


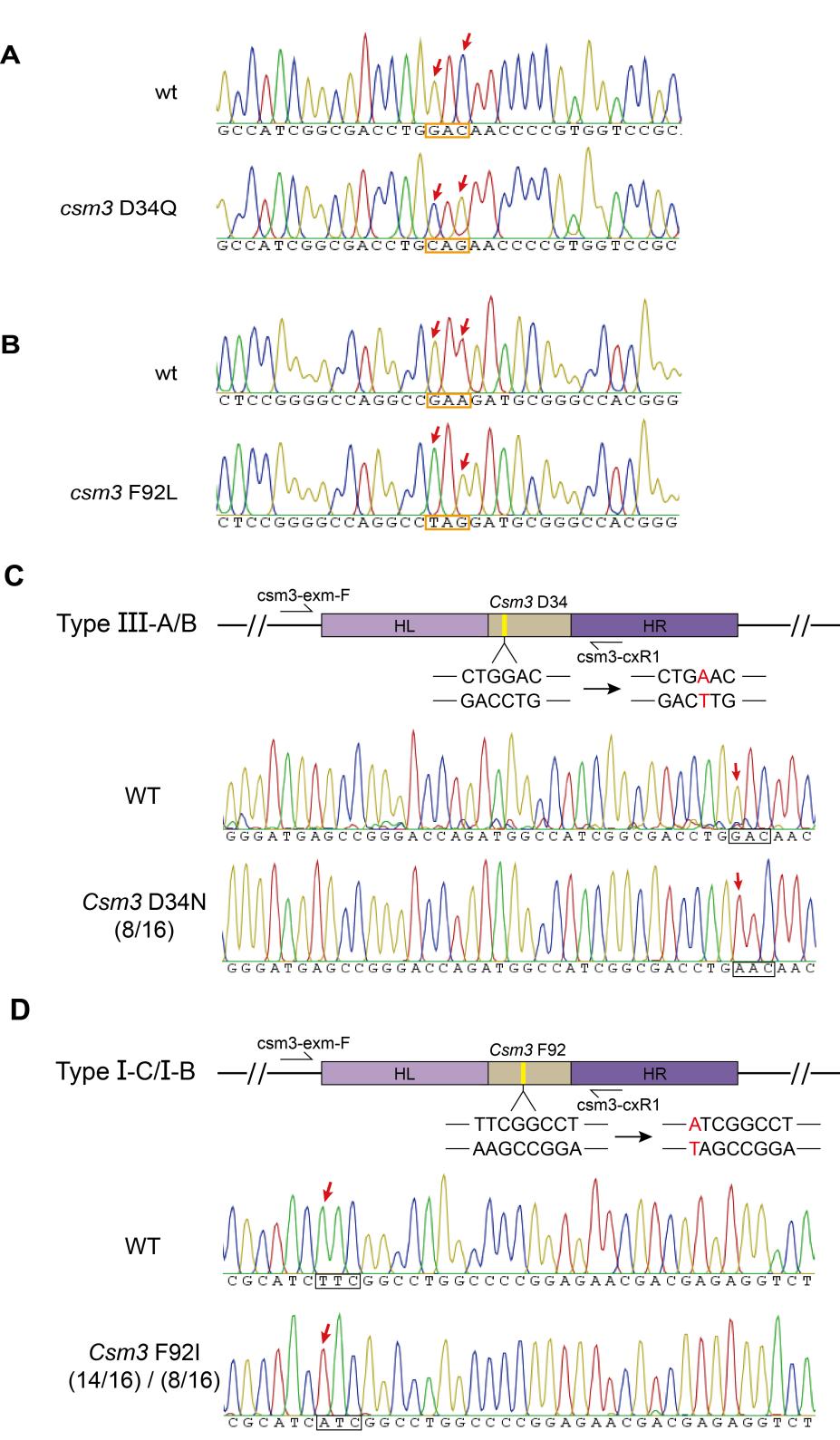


**Figure S6. The DNA sequencing results of *TTRS00960* gene knockout and** **integration.** DNA sequencing peaks of PCR products of the wild-type (wt) and the Δ*TTRS00960* (A), the Δ*crtB*::*TTRS00960* strain (B). Partial sequences of the *TTRS00960*, *crtB* gene and the recombination arm are presented.


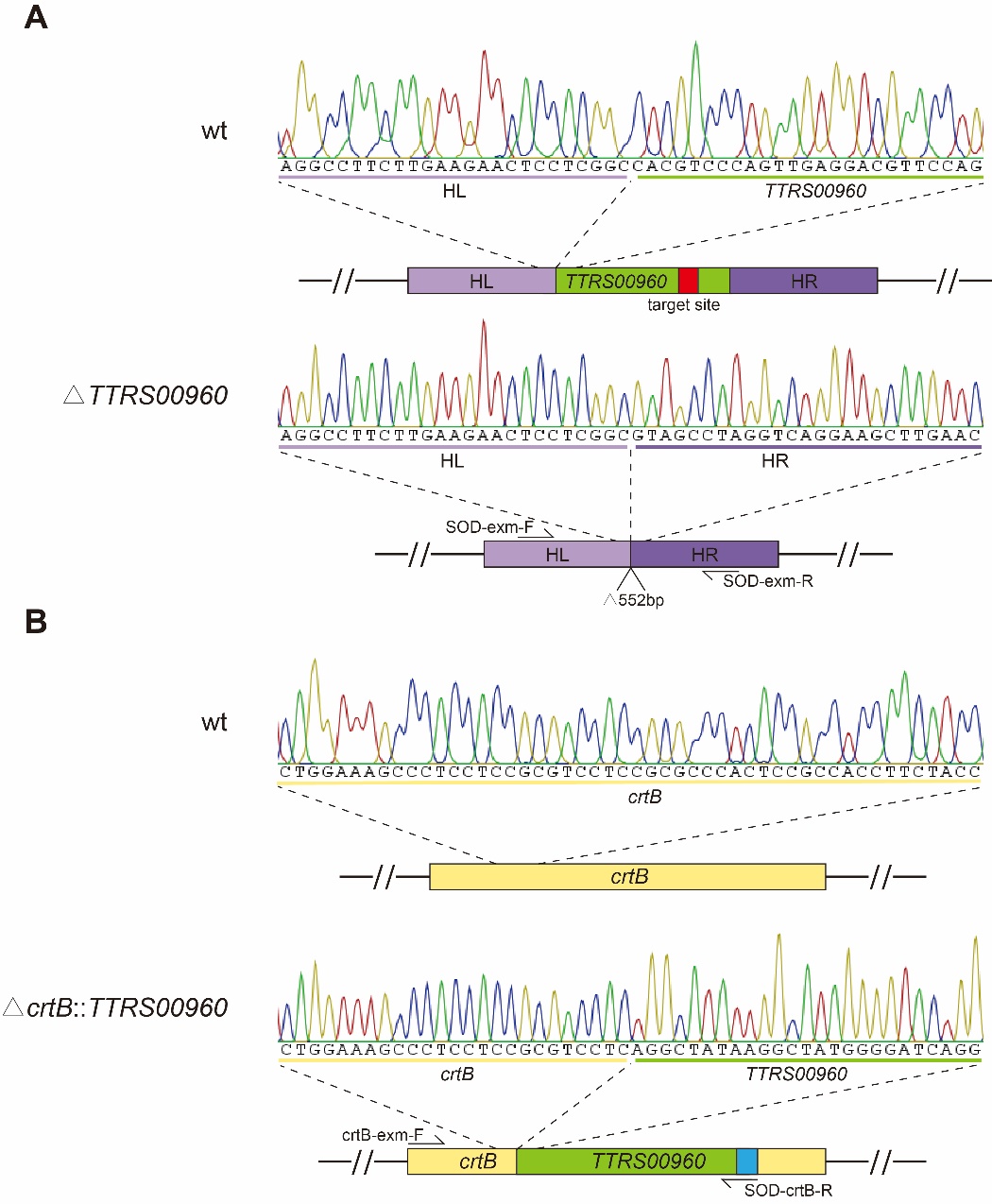

Supplement: Supplementary file 1 — Supporting information. [file MLF2-1-412-s002.docx]
