# Supplementary material for: High‐efficiency genome editing of an extreme thermophile Thermus thermophilus using endogenous type I and type III CRISPR‐Cas systems
Source: mLife. 2022 Dec 7;1(4):412–27. doi: 10.1002/mlf2.12045 (PMC10989782; doi:10.1002/mlf2.12045)
Supplement: Supplementary file 2 — Supporting information. [file MLF2-1-412-s001.docx]

**Table S3: Primers used to generate gRNAs and donors.**

| **Plasmid** | **Primers** | **Sequence (5’-3’)** |
| --- | --- | --- |
| pRKS-Kana-Sp1 | Kana-SpⅠ-F | CGACCACTTTCTCTAAGTATCCACCTGAATCATAAATCGGCAAA |
|  | Kana-SpⅠ-R | CAACTTTGCCGATTTATGATTCAGGTGGATACTTAGAGAAAGTG |
| pRKS-Kana-Sp2  pRKS-Kana-Sp3 | Kana-SpⅡ-F | AAACGTGTGCAAGGACCGACAACATTTCTACCATCCTTGAC |
|  | Kana-SpⅡ-R | CAACGTCAAGGATGGTAGAAATGTTGTCGGTCCTTGCACAC |
| pRKP31-crtB-Sp1-LR  pRKP31-KOcrtB-Sp1_1-7 | 1Rpt-crtB sp-F | CGACGCTCCGCCTCCGTCCCGAGCCGCACGGGGCCGAGGTCGGT |
|  | 1Rpt-crtB sp-R | CAACACCGACCTCGGCCCCGTGCGGCTCGGGACGGAGGCGGAGC |
| pRKP31-crtB-Sp2-LR  pRKP31-crtB-Sp3-LR  pRKP31-KOcrtB-Sp2_1-7  pRKP31-KOcrtB-Sp3_1-7 | 23Rpt-crtB sp-F | AAACCTGACCGACCTCGGCCCCGTGCGGCTCGGGACGGAGGC |
|  | 23Rpt-crtB sp-R | CAACGCCTCCGTCCCGAGCCGCACGGGGCCGAGGTCGGTCAG |
| pRKP31-crtB-Sp1-LR  pRKP31-crtB-Sp2-LR  pRKP31-crtB-Sp3-LR | crtB-L-F | TCCCCGGGAGGGGTGGTGACG |
|  | crtB-L-R | AGGCCTTGAGGACGCGGAGGAGGGCTTTCCAGTC |
|  | crtB-R-F | CCTCCGCGTCCTCAAGGCCTGGGAACGGGCCCTC |
|  | crtB-R-R | GGAAGCTTGGCCTCGAGGAAG |
| pRKS-ICcas3-Sp1-LR | 1RptIC-Cas3sp-F | CGACGTCGGTGGACCTTGCGCATCTTAGAGGGGCGGTTGGAGAA |
|  | 1RptIC-Cas3sp-R | CAACTTCTCCAACCGCCCCTCTAAGATGCGCAAGGTCCACCGAC |
| pRKS-ICcas3-Sp2-LR  pRKS-ICcas3-Sp3-LR | 23RptIC-Cas3sp-F | AAACTCCAACCGCCCCTCTAAGATGCGCAAGGTCCACCGAC |
|  | 23RptIC-Cas3sp-R | CAACGTCGGTGGACCTTGCGCATCTTAGAGGGGCGGTTGGA |
| pRKS-ICcas3-Sp1-LR  pRKS-ICcas3-Sp2-LR  pRKS-ICcas3-Sp3-LR | IC-Cas3-L-F | CGCGTATCGTAGCCCTCCTGATGGGCAG |
|  | IC-Cas3-L-R | GCCAGACGTAGAGGAGTTTGCTGGCCGCCTCACCCCTCT |
|  | IC-Cas3-R-F | GGCCAGCAAACTCCTCTACGTCTGGCGTGGGTCATAT |
|  | IC-Cas3-R-R | AGGTGGCGAGAAGTAAGCGGGAAACT |
| pRKP31-KOcrtB-Sp1_1  pRKP31-KOcrtB-Sp2_1  pRKP31-KOcrtB-Sp3_1 | crtB-L-F1 | CGCGTCGAACATCTCTGCGGAGTGTAGC |
|  | crtB-L-R | AGGCCTTGAGGACGCGGAGGAGGGCTTTCCAGTC |
|  | crtB-R-F | CCTCCGCGTCCTCAAGGCCTGGGAACGGGCCCTC |
|  | crtB-R-R1 | GCTAGACGAGAAGGGGGCCCATCAGGG |
| pRKP31-KOcrtB-Sp1_2  pRKP31-KOcrtB-Sp2_2  pRKP31-KOcrtB-Sp3_2 | crtB-L-F2 | GGAAAGGCCGGTCATGGCTTCC |
|  | crtB-L-R | AGGCCTTGAGGACGCGGAGGAGGGCTTTCCAGTC |
|  | crtB-R-F | CCTCCGCGTCCTCAAGGCCTGGGAACGGGCCCTC |
|  | crtB-R-R2 | GTTGTTGGGGTCCAGGACCACG |
| pRKP31-KOcrtB-Sp1_3  pRKP31-KOcrtB-Sp2_3  pRKP31-KOcrtB-Sp3_3 | crtB-L-F3 | AGGGCCTCCACGTCTTCCGCG |
|  | crtB-L-R | AGGCCTTGAGGACGCGGAGGAGGGCTTTCCAGTC |
|  | crtB-R-F | CCTCCGCGTCCTCAAGGCCTGGGAACGGGCCCTC |
|  | crtB-R-R3 | CCTCCAGGACCCAAAGGGCTC |
| pRKP31-KOcrtB-Sp1_4  pRKP31-KOcrtB-Sp2_4  pRKP31-KOcrtB-Sp3_4 | crtB-L-F4 | CCGGTCCCCAGGTCCTCGGGG |
|  | crtB-L-R | AGGCCTTGAGGACGCGGAGGAGGGCTTTCCAGTC |
|  | crtB-R-F | CCTCCGCGTCCTCAAGGCCTGGGAACGGGCCCTC |
|  | crtB-R-R4 | GCGTCCCGGTAGCGGCCGTAG |
| pRKP31-KOcrtB-Sp1_5  pRKP31-KOcrtB-Sp2_5  pRKP31-KOcrtB-Sp3_5 | crtB-L-F5 | GAGGTGCTCCAGGACGCCCT |
|  | crtB-L-R | AGGCCTTGAGGACGCGGAGGAGGGCTTTCCAGTC |
|  | crtB-R-F | CCTCCGCGTCCTCAAGGCCTGGGAACGGGCCCTC |
|  | crtB-R-R5 | GAAGGGGGTGTAGACCCGGT |
| pRKP31-KOcrtB-Sp1_6  pRKP31-KOcrtB-Sp2_6  pRKP31-KOcrtB-Sp3_6 | crtB-L-F6 | GGAGCCTTGCCCGGAGGAAG |
|  | crtB-L-R | AGGCCTTGAGGACGCGGAGGAGGGCTTTCCAGTC |
|  | crtB-R-F | CCTCCGCGTCCTCAAGGCCTGGGAACGGGCCCTC |
|  | crtB-R-R6 | GAAGCCCCGGGTCTTCCCGG |
| pRKP31-KOcrtB-Sp1_7  pRKP31-KOcrtB-Sp2_7  pRKP31-KOcrtB-Sp3_7 | crtB-L-F7 | TAGGCGGCGAGCATGGCCC |
|  | crtB-L-R | AGGCCTTGAGGACGCGGAGGAGGGCTTTCCAGTC |
|  | crtB-R-F | CCTCCGCGTCCTCAAGGCCTGGGAACGGGCCCTC |
|  | crtB-R-R7 | CCGTCCAGCCGGTCCCGCT |
| pRKP31-csm3 D34Q  pRKP31-csm3 D34N | 1Rpt-csm3-D34-F | CGACTTGTCCAGGTCGCCGATGGCCATCTGGTCCCGGCTCATCC |
|  | 1Rpt-csm3-D34-R | CAACGGATGAGCCGGGACCAGATGGCCATCGGCGACCTGGACAA |
|  | csm3-D34-L-F | CGTCTGGGTCCAGTGGGCGCT |
|  | csm3-D34-R-R | GGCTCCTGACGGAACCTCTCT |
| pRKP31-csm3 D34N | csm3-D34N-L-R | TTGTTCAGGTCGCCGATGGCCAT |
|  | csm3-D34N-R-F | GCCATCGGCGACCTGAACAACCCCGTGGTCCGCAAC |
| pRKP31-csm3 D34Q | csm3-D34Q-L-R | CGGGGTTCTGCAGGTCGCCGATGGCCATCTGGTCCC |
|  | csm3-D34Q-R-F | GACCTGCAGAACCCCGTGGTCCGCAACCCCCTCACG |
| pRKP31-csm3 F92L-Sp2  pRKP31-csm3 F92L-Sp3  pRKP31-csm3 F92I-Sp2  pRKP31-csm3 F92I-Sp3 | 23Rpt-csm3-F92-F | AAACGGCCTGGCCCCGGAGAACGACGAGAGGTCTTTGGCAGT |
|  | 23Rpt-csm3-F92-R | CAACACTGCCAAAGACCTCTCGTCGTTCTCCGGGGCCAGGCC |
|  | csm3-F92-L-F | GTCTGGGTCCAGTGGGCGCTTT |
|  | csm3-F92-R-R | GGGCTCCTGACGGAACCTCTCT |
| pRKP31-csm3 F92L-Sp2  pRKP31-csm3 F92L-Sp3 | csm3-F92L-L-R | AGGCCTAGGATGCGGGCCACGGGGTCCTTGGGGTC |
|  | csm3-F92L-R-F | TGGCCCGCATCCTAGGCCTGGCCCCGGAGAACGAC |
| pRKP31-csm3 F92I-Sp2  pRKP31-csm3 F92I-Sp3 | csm3-F92I-L-R | TTCTCCGGGGCCAGGCCGATGATGCGGGCCACGGGGTCCTT |
|  | csm3-F92I-R-F | TCATCGGCCTGGCCCCGGAGAAC |
| pRKP31-csm3 10xHis-Sp1 | 1Rpt-csm3-His-F | CGACCCTAAAGGACCACTTCCTCCACCTTGAGCCTTTCCTTTAG |
|  | 1Rpt-csm3-His-R | CAACCTAAAGGAAAGGCTCAAGGTGGAGGAAGTGGTCCTTTAGG |
| pRKP31-csm3 10xHis-Sp2  pRKP31-csm3 10xHis-Sp3 | 23Rpt-csm3-His-F | AAACCTCCACCCCGAAAGGCTTACGGAGGACCAGGAGGGCTG |
|  | 23Rpt-csm3-His-R | CAACCAGCCCTCCTGGTCCTCCGTAAGCCTTTCGGGGTGGAG |
| pRKP31-csm3 10xHis-Sp1  pRKP31-csm3 10xHis-Sp2  pRKP31-csm3 10xHis-Sp3 | csm3-His-L-F | ATGAAGCTCAAGAAGGTGATCC |
|  | csm3-His-R-R | GGTGGCGTAGGCGTTCGGCTCCTT |
| pRKP31-csm3 10xHis-Sp1 | 1Rpt-csm3His-L-R | ATGATGATGGTGATGGTGATGGTGGTGATGAAGGACCACTTCCTCCACCTT |
|  | 1Rpt-csm3His-R-F | CATCACCACCATCACCATCACCATCATCATTAGGGGGCCTGGATGCGGGCGAC |
| pRKP31-csm3 10xHis-Sp2  pRKP31-csm3 10xHis-Sp3 | 23Rpt-csm3His-L-R | ATGATGATGGTGATGGTGATGGTGGTGATGGAAGTAGACCTGGCCGTAGC |
|  | 23Rpt-csm3His-R-F | CATCACCACCATCACCATCACCATCATCATCTCCACCCCGAAAGGCTTACG |
| pRKP31-TTP0220 Sp1- LR | (I)P0220-SP-F | CGACCCCTGAGCCCCCCGTAGCTGTAGACCTCGTCCAGGACCCG |
|  | (I)P0220-SP-R | CAACCGGGTCCTGGACGAGGTCTACAGCTACGGGGGGCTCAGGG |
|  | P0220-L-F | CGGCCCTCCTCCTCTTCCTGC |
|  | P0220-L-R | CTCGGGGGGCACCTTGGCCTCGCTTAGGGCGAAG |
|  | P0220-R-F | GCCCTAAGCGAGGCCAAGGTGCCCCCCGAGAGGGTC |
|  | P0220-R-R | CTCCTGCCGCAGGAGCCTGCC |
| pRKP31-TTP0222 Sp1-LR | (I)P0222-SP-F | CGACCCCTTTGCGCCATCTCCTCCGAGTAGCCCGCGAGCCACTG |
|  | (I)P0222-SP-R | CAACCAGTGGCTCGCGGGCTACTCGGAGGAGATGGCGCAAAGGG |
|  | P0222-L-F | CCGCCCCCCTGGACCGGATC |
|  | P0222-L-R | GTACTCCACGCTGAGTACCCCCTCCAGGCTCCAC |
|  | P0222-R-F | AGCCTGGAGGGGGTACTCAGCGTGGAGTACCTGGA |
|  | P0222-R-R | TAGGGCGATGCTGGCCGTGAG |
| pRKP31-TTP0042 Sp1-LR | (I)P0042-SP-F | CGACCGGCGAGGCCGTGGCCCAGGAGGAGGTGGTGGGCGGCGCG |
|  | (I)P0042-SP-R | CAACCGCGCCGCCCACCACCTCCTCCTGGGCCACGGCCTCGCCG |
|  | P0042-L-F | GCGCCTCGTGGGCAGCTACGG |
|  | P0042-L-R | GCTGGGGAAGTCCACGTAGGCGCTGGTGGCCACT |
|  | P0042-R-F | GCCACCAGCGCCTACGTGGACTTCCCCAGCCAG |
| pRKP31-TTP0042 Sp1-LR | P0042-R-R | GCCAGAACTCCAGGTCCACCC |
| pRKP31-TTRS00960 KO- Sp2 | 2Rpt-SOD-F | AAACGCCTACTACCTCAAGTACCAGAACCGCCGGGCCGATTA |
|  | 2Rpt-SOD-R | CAACTAATCGGCCCGGCGGTTCTGGTACTTGAGGTAGTAGGC |
|  | 2Rpt-SOD-L-F | CTTCCTTGGGAGAGGCGGTCTTGGT |
|  | 2Rpt-SOD-L-R | GCCGAGGAGTTCTTCAAGAAGGCCTGAT |
|  | 2Rpt-SOD-R-F | TTGAAGAACTCCTCGGCGTAGCCTAGGTCAGGAAGCTTGAACGGGT |
|  | 2Rpt-SOD-R-R | GTGGAGGCCCTCACCATGGTGGTGGT |
| pRKP31-TTRS00960 integration- Sp2 | 23Rpt-crtB sp-F | AAACCTGACCGACCTCGGCCCCGTGCGGCTCGGGACGGAGGC |
|  | 23Rpt-crtB sp-R | CAACGCCTCCGTCCCGAGCCGCACGGGGCCGAGGTCGGTCAG |
|  | TTRS00960int-L-F | GAGGTGGTAGGCGGCGAGCATG |
|  | crtB-L-R-SOD | CATAGCCTTATAGCCTGAGGACGCGGAGGAGGGCTTTCCAGTC |
|  | SOD-crtB-F | AGGCTATAAGGCTATGGGGATCAGG |
|  | SOD-crtB-R | GCGGCAAGGGGCTTTGTGAGGAAG |
|  | SOD-crtB-R-F | CAAAGCCCCTTGCCGCTCAAGGCCTGGGAACGGGCCCTC |
|  | TTRS00960int-R-R | GGAAGCCCCGGGTCTTCCCGG |

**The sequence of the endogenous CRISPR systems (5’ to 3’)**

CRISPR-11: gttgcaagggattgagccccgtaaggggattgcgactttgggtgcgagggggtggaccgctagttgcgcctctagttgcaagggattgagccccgtaaggggattgcgaccggactcacaaggacggtgcggagctggcggtcctccccagttgcaagggattgagccccgtaaggggattgcgaccgggagccgccgcctcgaagagggtctcccaccactgttgcaagggattgagccccgtaaggggattgcgacccccccacaccgcgccccacttcacaaaacatagcgctatagtgttgcaagggattgagccccgtaaggggattgcgacctgtccttggcttccctgaggagcctttcagcctcaaaaagccgttgcaagggattgagccccgtaaggggattgcgacatgaagatgcggatgattgcaaagagccagataggttgcaagggattgagccccgtaaggggattgcgacggcaccgtggcttgtgccttgcgcatgggcaagcggggtgttgcaagggattgagccccgtaaggggattgcgaccccacccagccggggcaccgtagcttctgcctcgcgcatgggttgcaagggattgagccccgtaaggggattgcgacgcgcccatgagcacctctttcgccgcggtcctagtcatcctagttgcaagggattgagccccgtaaggggattgcgacgacacctgggagaggcggagggagtggattaacttcggggttgcaagggattgagccccgtaaggggattgcgacccccgtatagttcccgccagatgttggcgagttcctccgctggttgcaagggattgagccccgtaaggggattgcgaccgctggaccttcctcgccacctgcctcaaaacgtcccagggttgcaagggattgagccccgtaaggggattgcgaccctaggggaagaaggactcgaggtgctccttcacgaccttgggttgcaagggattgagccccgtaaggggattgcgactcagcccccctagcctcctcgagggccttctgcccctcgttgcaagggattgagccccgtaaggggattgcgaccctctcgcacaaggggacggataatctccccgttgagcgagagttgcaagggattgagccccgtaaggggattgcgaccagtatgcgtaggcctcggctatcctctcgtacagcgcccagttgcaagggattgagccccgtaaggggattgcgacccagggaaatcatgagaaccgacacggttggacagggttgcaagggattgagccccgtaaggggattgcgacccctagggtgcgggctaggggctaggctaagcctagccctagttgcaagggattgagccccgtaaggggattgcgac

CRISPR-1:

gttgcaagggattgagccccgtaaggggattgcgaccgcgatgacgatgcagggcgccgagtgaaacgttaaaggttgcaagggattgagccccgtaaggggattgcgacatcaccttcttaggtgtcgggttcctttttgccgttctaacgttgcaagggattgagccccgtaaggggattgcgacaccaagatgtagtcatccccaaagtccttgacgagcttagtcatgttgcaagggattgagccccgtaaggggattgcgcc

*cas2* (III-A):

atgggaaagcgtctctatgccgtggcgtacgacattccggacgacactcgccgggtgaagctggccaacctcctgaaaagctacggggagcgggtccagctctccgtgtttgagtgctacctggacgagcggcttctggaggacctgcggcggagggccaggcggcttttggacctgggccaggacgccttgcgcatctaccccgtggcgggccaggtggaggttctgggcgtggggcctttgccggagctccgggaggtccaggtgctgtga

*csm1* (III-A):

atgcttggggacggcctgagcgtggccctggcgggacttttgcacgacgtgggcaagctctattcccgcgcccgctggggcgagcgggacgatcgggttcccgaccgcacccacaccgcctacactgcccacttcttccgggagcatgccgggctctttcgcggggcgggcctggacccggactggctcgcccggacggcaagccgccaccacgagggctggcgggaccgcccccagtaccggcccgagactcccgaggagtggtgcgtggccctggcggacacctacgcctccaaggagcgggaggagggggagggtgggggaagcccccccgaggtgcccctttcccctcccttccgcaggctcctcctggggggcgaggaggggagggaaggagggtacagccccgtgggggccggggggcgggtgggcctcgaggccggggggctttacccggaggagcggcccaacgtctccaaagacgtctacaagcggcttcttgagcggctggaggggcggcttgaggagatggcccgcttctccctgggcaaggaggcccttgtcctgaacctggccctggccctccaggagaccctttccctggtgccctcggacacccagtcggagccggacgtctccctctacgaccacctgcgcctcacggcggccatcgcccacgccttgtggcttttccacgggggaagcccctcggcgcaggacctgcgccaggacggggagaagttcctcctggtggtgggggacatggggggcatccaggggcacatctaccgcatcgccggggcggaggcgggagtggggggcatcgccaagcggcttcgggcgaggagcctcgaggtgagcctggccgcggaggccatggccctggggcttctttggcgcctgggcctcacccccctgaaccgcatcttgggggcggggggcaagttctacctcctcctgcccaacaccgaggaggcgagggcggccctggaggggaccagggaggcctggggtaggtgggccctcaagcgggggggaagcctcgtgccccacctggcctgggtggccttccgggggcaggacttccgggacttcgccgccctcctcaagcgcctccacgaggccctcgcccgggagaagctcaggcccttcgccttcctcgcctccacggggggggtcctgggggcgcccctccgcccctgcgccgcctgcggcctggagcccgcccgaagggacgagcccggaagcctctgcccggactgcgagcgggaggcggccctcggggcccggcttccccgaagcgaccgggtgggcttcttcctggaggaggctccgaggccctacctggactttcccggcctgaaggtgggcctgggggggccgttggagggggccttccacctcttccgcgcccggctggacttcgccccctggcccgacccctcggaggccaagcccctcctcggccacctgccccgggtggagcacgccctgaaggccaaggggtggagcctggaggcctaccgggcctgggccgaggaggaggggcttttggaggacgaggagccccatcccgaaaaggtcctcaccttcgccgaactcgccgccctctcggagggcgccccctacctcggggccctcatgctggacgccgaccgcatgggggaggccttcgccacaggcttccgccgcgaggggcgggacctcgccaccccgagccgcctcgccgccctctcccgcaccctggaggtcttcttcaccacggaggtcctcaccctcctggaggagccccggcgctaccgggagcggcttgggtgggacgacctcgaggcccagggcaaggaggcccgctaccccctcctctacagcgtctactccgggggggacgacctcttcctcctcgggccctgggacgcccttttggacttcgccctggacctggagaggctctaccgcctcttcacccgccacccgcggctcaccctctccggggggttcctcctcgtcccgccgagcctccccgtgcccgagctcgcccggcttttgggggaggcggagaagcgggccaaggccgaggggcgggagaggcttttcctcttcggccaggcggtgccgtgggagaccttgcggggccttcgcgcctgggccgagggcctgcgccaagacctgcgggccgagcgggtgagccgggcccaggtctaccgctggctcctcctctggcgcaggttctcccccctggaggaccccggggagcggatgcgctacaagcccctcttggcctacgccctaaggcgcgtgcgggagcgggacgagggggcgtgggaaaggtacctaaagcttcttgaccaccaggatccggcctggacctaccttcccgtctgggtccagtgggcgctttaccgggaaaggagggtgtga

*csm2* (III-A):

atgccggcgttggagtttttcaaggacaaggagcggggagttctggaccctaaggtctttgagcgggcccgggaggtggcggaggggctcgccagggggaagctcaagtccagccagttccgcaactacttcgccgagctcagggccctggagaaccgctttgagcgggagaggcgcaaggaaggggaggagttggccttcgcccggctcgtcccccagcttgagctcctcagggccaagctcttctacaacacccgctcccagggccccttgagggacgccaaggagttcgtggagttcatggaggaggccctcgaggcggggaagcggagcccaaaggactttgaagcgatgatgaagtacgtggaggcggtgctcgcctacttctacgccttggcgaagtag

*csm3* (III-A):

atgaagctcaagaaggtgatccgcatccgctcggtgctcctggccaagacgggcctcaggatcgggatgagccgggaccagatggccatcggcgacctggacaaccccgtggtccgcaaccccctcacggacgagccttacatccccggctccagcctcaaggggaagctccgctacctcctggagtggagcctgggcggggactacatcctgaaggccaaggacaagcacgtctacgcctcccccgaccccaaggaccccgtggcccgcatcttcggcctggccccggagaacgacgagaggtctttggcagtggcccgggagcggggccccacgaggctcctcgtgcgggacgcttacctcacggaggacgccaaggaggccttagagcgcacctccgccaggggcgggctctacacggagatcaagcaggaggtcttcatcccccgcctgggcggaaacgccaacccccgcaccacggaaagggttcccgccggggcccgctttagggtggagatgacctaccgggtgctggacgacctggacgaggagtacttcgggaaatacctcctccgggccctggagcttctggagctggacggcctcggggggcacatcagccggggctacggccaggtctacttcctccaccccgaaaggcttacggaggaccaggagggctggcccctaaaggaaaggctcaaggtggaggaagtggtcctttag

*csm4* (III-A):

atgcgggcgaccctcttccgcctctacttccagggccccctcaaggccctcccccgggcccccaccctcctgggccacctcttctggtggtaccggtacacccacggtcgggaagctttggaggagcttttagagaggttccgtcaggagccccctttccgcctctccagcgtctaccccgagggctggcttccccggcccaagttgcctccggtgcaggtggaggaaaccaccctgcggaaggccctgaagagcctctccctggtgcgcctggacactttccaggccctggcggaaaggggggaggaggccctcctcgaggcccccgaggtccagggaaaggccaggcccccggagatgcggaggctccgccgcacccgggtgggggtggaccgggccgcgggcacggcccgccccggcgtcctcttcacccaggagtacctcttccccgatcccaggaccccctatgccctttacgtcctgggggaggccccctttgacctcggggaggccctcgccttcgtgggggaaatgggctacggggggcaggccagcctgggcctcgggcgcttccgggtggaaggcccctttgcggtggagcttcccgaggccaaggagccgaacgcctacgccaccctggctccggggcccttggaagaggccctctactacgaggtagagccttactggggcaggctcgggggggcctacgtgggggcccggcccttcaaaaggccctacctaagggccaaggaggggagcgtctaccgggggcccacccaccgcctgctggaggtgacccccacggagcccccggaggcggggacgcgggtgtgggaggccctcgtggtctttcccttgggggtgagggtatga

*csm5* (III-A):

atgaggttcctgaggagctttcgcctggagctggaggccctaagccccgtgcacgtgggcaccggggaggcctaccccgcctacgcctacgtgccggacttcgccaggaaggcggtccacctcctggaccccgcggccctcctcctcgccctccccgaggctaggcgccgccagtacctggagaaggtggcccagggccccaaggcggcccaggaggtcctgcgctatctcctggaagaagaccagcttccccgggaggccgtcctccacaccctccccgccagcaaggcctttttggaagctcttcgctccgccacggaggaggccctcctggagtaccgcccccttccccgttcccccctgggggcctacctccccggttccagcgtcaagggggctttgcgcaccgcctggctcttccacgtcctggtgagggagggcaaggtggcggtctttgaccggaaggagggggtgtggcggctcagggcctggcgggagggggacgggggcacccacgtctacccctcccggaaccccagcctttacgagaaccaggcctttgagggcgcggtcctgggctacgcccgggaggggaaagggaggcggatgtccttggacctctaccgcgaccccttccgggcggtgcgcctttcggactcggggccggtggagaccttcttgaaccgcctcggggtcttccatcctcacaaggacacctcgaggatggtcctcttggcggagaccttccgcataggcacccgcttcgccctcaccctccgctaccacgagggccttagccgggacggggatggggagcggggggtgtccatgcccattcccccggaggacttggtccgggccctcagggactactacgggaaggtggcggagtgggaggagggcttcgccgaggagcacaggcttaagcgggccctggaggtctaccgggccctaagggagcggcttcaggacccggaggccttccccctccgggtgggctttggctcggggaggctcgccctgcgtctcgccctcctccttcccgaggaccaccccgaggcccaggagcccaagacccggaagaccgcgggggcccagaaccctgtggacggctaccccttaggctggatggtggggaggctagagcccctctag

*csm6* (III-A):

atggaggaccttgacgccctttgggagcggtaccgggaggcggtgcgggcggggggcaacccccaggccctctaccaggagatggtctggcctgccctcctcgccctctggcgggagaagccccgggtctacccctttccccaggccttcgccgtgtccgtgcacaccctggggacgagccccgaggccacggccctcgccatcttgggagcgggggcggagcgggtctacgtgctccacaccccggaaagcgcccgcttcctccccaggcttcgccaggacacggggaaggacctctaccccgtggagatcggcaagagcgacgtggaggccatctaccgggaggtgaagcggcttttggagaagcacccggaggtgcccgtggccctggacctcaccagcggcaccaaggccatgagcgcggggcttgccgccgcgggcttcttcttccagcgcttttaccccaaggtgcgggtggtctacgtggacaacgaggactacgaccccgagctccgccgcccccgggcgggcacggaaaagctccgcatcctccctaacccccacgaggccctggcggaggtggacgccctcttcgccaaggagctctacgggaagggggagttcggccaggcggcggcctacttccgcggcatggtggggaggacggggaaccaggcctacgccctctacgccctcctcgcggagatgtaccgggcctggcgggccttggacttcggcgaggcgttgaaggcggggaggaagctccttggccagctctcccagaacgtctggctgaaccatcccctgaacgcccggagggaggccctcgaggcccaggtggccctcctcgaggccgtgggccgcttcctgaaggcccgggacttcgcccttaaggagggggtgtacggcctcgcccgaaccctcttgcacctcgcccaggaggccaaggaagaggccgccgtcctcgcggccctctacgcctaccgggccctggagctcctcctgcaggaaaggctcgcccttctcggcaggcgggccgaggccccggggctttccccggaggaggcggaggccctgcggaaggccctggcggagcttcttggggtctcgcccgaggaggtgcgccttccggccaagctcggccttttggacctcctcgccttcctccgcctcaagggggatgaggcgcttggccgcctttctttggcggagcttcggggcctcgccggggcgctcaaagggaggaacagcgccctcctcgtccacggctttgacgtgccctcgcccaaggcggtggaggggatcgcccgcctggcccagggccttctccaggacctcgaggcccggaccgctctcggccccctttccccggagcccgtccccctggggttttag

CRISPR2:

gttgcaagggattgagccccgtaaggggattgcgactggggtatatcctctttatccccggtattcttgacaggtctgttgcaagggattgagccccgtaaggggattgcgacttcacaaaatcgtacttgaggcaagcgaaagcgacgagttgcaagggattgagccccgtaaggggattgcgacccctgagggggaggaggaggtggcttgcgggtaaaaaagttgcaagggattgagccccgtaaggggattgcgacttcgaagcgccccgccccgaggcacttcccacaccctttcccgttgcaagggattgagccccgtaaggggattgcgacgtgctccttcaaactcctctccagagcctcctggatcagttcatgttgcaagggattgagccccgtaaggggattgcgacactccctctacgccccctaactcgcggtacgcgaagaggggttgcaagagattgagccccgtaaggggattgcgacgacaggtaagggtgtagaggtcgtccagcttcataagttgcaagagattgagccccgtaaggggattgcgacaagtgaggatgtacccccgccccttgagcttcttctcgttgcaagggattgagccccgtaaggggattgcgacctttgcgccgcgcggcctagcgccgaccgcgtatcccgttgcaagggattgagccccgtaaggggattgcgacctttaagggagtcgccttcgcccacgtcttccttggtgttgcaagggattgagccccgtaaggggattgcgacgcgaggagccgccgcaccccgatgatgcgcccctggtcgttgcaagggattgagccccgtaaggggattgcgactccttctttgggccagctgatgccccaggcaagccccggtgttgcaagggattgagccccgtaaggggattgcgacgcttctccgttgcgctgagttcgccgttgctccagacatgggttgcaagggattgagccccgtaaggggattgcgacgtgtggccgcggctgcgcgccgcgcggcccttgcgcccatgcgttgcaagggattgagccccgtaaggggatggcctc

*csx1* (III-B):

atgcaagccccggtgtacctgtgccttctgggcaacgacccggccccggcctacttgggcttgaaggtggtggagcgggaggcggggagggtggcgaaggccgtcttctactccttcccggcgtggaacgaggagtacgggaaaaagcgccaggccttcttccgcctcctttccgaaaagggcgtcctctacgaggaaaggcccctagaaaagggactggaagaagcggaggcccgggaggtctgggtgaacctcacgggaggggccaagtactgggcggtccggttcctcgggcactggcggcggcccggcgcccgggtcttcctcgtggagggccaccgcgccctcgaggcgcccagggcccttttcctctggccccgggaggaggagcgctcccttgaggccgaggccctcaccctggaggagtacgccaggctctatctggagcccctgggggaggcctgggagcgggtttccccgcccggggccttcccccctggggctcaggcggcccgccttcccggccgggagggcggggtgttcgtggtccaccggggccttccctactggtactgggtgcgcccccatctggggggcgaggccaaggaaatgtcccggaaggccctttccgccttctccggcgaggccaagcgtctggggggccagctctgcctgcccgtggtcccataccacaaggcgcacctccgctcccgccaccctaaggagcgggaaaacgtcttcgcccgctggagggcctgggcccgggagtacggggtgttcctggtggacccgggcaggcctttggaagaggaggtggcttccctcatcaaggggaaggcctccaagaaggccctgcccctgccccaggaggggccccttctcctggccctggtttccgagcaggccgttcccctctatgcggcctacctccacgccggccccagggaggtctacctcctcaccacccccgagatggaaagccgcctccgctgggcggaggccttcttccggggcaagggggtgcgggtgcaccggagcttcctttccgggccctgggctttgcgggaggtgcgggacctcctcgcccccgtggtggaggaggccctgcgccggggccaccccgtgcacgccaacctgaacagcgggaccacggccatggccttgggcctctacctggccctgcgggatggggcccgcgcccactacctggatggggaccgcctcctccttctggacgggggggaggcggaggtgccttgggaggagggaaggcctgaggacttgctcgccttgcgggggtaccgctttgaggaagagtaccccgacgcccggcccgaccccgggctcctcgccctggccgaggagatcctgaggcggtgggacgaggtgcagacctcctgggaggcctcccccttggtgcggcggttcctgaagttctggaaaaagcgcttcggccaggccttccccccgaagcgcctttccaggctcaaggggcttcccctggagtacgcggtctacagccacctgaacgcccacctggccccaaaaggaggccaggcccgcatgggcgggcacctggtgcccctggggggcaatgaggccctggccccccagttcaccgaggtggacggggtcttcttccaccggggcgccctctggttcgtggagtgcaagcccacggacgagggtctgcgggagcgggcccctatcatggccgagctggtgcgctccgtgggcggggtggaggccagggggctcatggtggcccggcgttggcggggagccccgcctccggcgagccccaacctggtctacatggccctggagggcggggagggcgtgggggtctaccgcttccccgaggagctggaaaaggccctctcacggaatccggcgccgcggaggggctag

*cmr2* (III-B):

atggagcaccttctcgctatcgccctgggcccggtgcaggagttcatcgccaccgcccgcaggacccgggacctctacgcgggaagccgcctcctctccgaggcggccgcccgcgccgccgaggccctggcccgggaggtgggggcgaaaaacctcattttccccgcccccgaggacgaggcggggctggagcggctggccggggcgggcatccccaacgttcttttggtccgggttccggaggggaaggaccccaggggcctcggggaacaagccttgggagccgcccgggactacctccgggagagggcggaggaagttctggggccgcgcagggacctccttttctggagggaggccctggcccaggtggaagacctcctggagggctactacgcctaccttcccttggagggcgactatccccgggcccgggagcggctcatggccctcctggccgcccgcaaaaacacccgggacttcgcccccgtctcctggggaagcccggcctacaagagctccctggacggggcccgggagagcgtcctccgcctgcccgaaagggaggcggaccacctcagggtgcggctcggcctgcgccccggggagtaccttgcggggcccgacctcctcaagcgctggtggaaggcggggcacggcttccttagcaccacccacatggcggccctgcccttctgggagggggtcaggcgggcgggcctggaggcggtcctaaaggaggccctggaggagcttgggggcctcgtgggggaggaggcgcgggcggaggtccgccacccggttttgcgggacacccccttcggggagtgggacgtgcgcctcctctacgagagccgcctcgaggagtttccctccctcgccgaggaccccgggcttctggagaaggcgcgggaccgcctgagggcgctttggcggaggctttcccccaaggtgagggttcccccgggcgcctactacgccctcctccacgccgacggggaccggatgggggagaccctggacggccttccgtcccccgaggcccaccgccgcttctccaacgccctggccctggggttcgccgcccaggtcaaggacatcgtggaggcccacgggggcgggctcgtctactcggggggggacgacgtcttggccctgcttcccctccacacggccctcatggccgcgagggccctggcggaccgcttccgggaggccatggcccccttcggccgggagggccgggcgccgagcctctccgtgggcctcgcggtggtccaccacctggagcctctgcaggacgccctggacctggcccgccgggcggagaaatgggcgaaggagggcgagcccaagcgcaacgccctctgcgtggcctacagcccccgctcgggggcggagcgcctggtccggggccggtgggacgagaacccccccctcacccgccgcctcctccgctacgccgacctcctgcgggcgggggaggtgccttccagggccgcctacgagcttctggccctggtccgggaagcgggggaggccctccccggggaggccctggtggccgaggccctgcggatcctggggaggaaggagatgaagcgggcctaccgggaggagctcgaggcctggctccggaccggggaggacgtgcgccgcctggcggaggagctcatcctcgcccggcccttcgccgaggccctggaccaggcgggcgtacccgtggagagccgggaggtgtgggatgctcattga

*cmr3* (III-B):

atgctcattgagcccagggatcctttgatcgtgcgggacggcaggcccttcaccaacagccccggggcccgggccaagagccttcccttccccctgccccagaccctggccggggcctaccgcacccgccgcgccctcctggagggccttcctctccccgagagggcggaggaggtcctccggtggggcctcagggggcccctcctcgccgaggagggggaaggggggtggcggctcctggcgccccggcctctggacgccctaaagctcggggaggccctctaccccctccggcccctggagcttccccaaggggccgggaccaacctgccggaggggctttctcccgtgggtcttcccagcccggccctcaaggagaagcccgcccccctgcccgccttctggtactgggagagcttcctggaatggctccttcaagacgccccggcgggtttcgcccccagggggcacgaggggcccgtcccggagacgcgcacccacgtggccctggaccccgcggcccagacggcccgggaggggttcctctttcagacctcggggctggagtttgtccgagggaggcgccgcctcgccctggtgctctggcccgaggggccggagcccgagggggtcttccccctggggggggagaggcgcctcgccttctggcagaaaggcgggcccggggttcctcccctgcccgaggaggtggtggcggggctcctccgccacagggcggcccgcctcgtcttcctcacccccgccttcttgggggaggcttacctccccaaggggagggccttccaaggggcttccgtggtggccgcggtggtggggaggccggttgcggtctcgggctggaacctcaaggaggggaagcccaagccgagccgccgggccgtgcccgcggggagcgtctacttcgtccggttcccggaggcctggggggaaggggaggtccgggattgggcggggaaggtctggttccagaacctctccgaggaggagcaggaccggcgggacgggttcggcctggccgccctgggcctttgggacgggaagcttaggcgctgggaggaggcatga

*cmr1* (III-B):

atgagaagggcgtttcgtgaggcgttctcgccgaggcgaaaggaggagggagaggtcctggtgcgccgggacggcgcgcgggtcctcgcctgggagcgcacgtacacgctcctcacccccctcttcgggggcggggtggagcctagggaggcggatccggtgagcgtggtccgggccacggcggtccgggggcacctccgcttctggtggcgggccgtgagggggtggcgggcggggggttcgctggagaggctttgggagctggagtccgccttgttcggccacgcgggggagggcggggcctcgcccttgagcgtggaggtggaggttctaagggaaggggaaaaggtaggcatcgcccagtacggccgcgcagtccagtggtacctgggctttcctctacgcggggacaaggaatgggccccggtcaaggagggcgtggccttccgcctccgcctccgctttcccgaaaaggtaggggagctgaacttctgggaggagctcgaggccgccctctgggcctgggagaccttcggggggatcggggcccggacccggaggggctttggcgccctcctgccccagggggccggggtgccgggggaggaggagatccgagagaagcttcgccagtatagccaaaaagcagggtggcccgaaggggtgccccacctcaccccaaaaagcttcgtgcgggtggtgcccctctcctggaaagaactcgccgagcgctaccaggccttccgccaggcccgccccgggggggaccccagaagcccgggccgctcctactggcccgagcccgacggggtgcggcgcctcacggggcggcacgccccccaccacctgcccaggcaccccgtgcacaagttcccccgggcccacttcggcctgcccatcatcttccacttcaaggacaggggcgacccccccgacaccaccctccggctcaaggaggccgaccgccgggcgagccccctcctcttccgcccgcttggggaagggcagaagccctgcgtggtggcggtcctcgagggggccaggttccctggggaaaagcttgttttggagggcaaagacggccgcacttgggacgtggacccctggctcacccccgaggaggcccaaaagatcccggtgctggggggtgaggcggaccccgtcttggccttcgtgaaaagcttgtag

*cmr4* (III-B):

atgagccacgttgcgcttcttttcctgcatgccctctcccctctgcacgcggggacggggcaaggcatcggggccatagacctgcccatcgcccgggagaaggccacgggcatcccctacctaccgggaagctccctcaagggggtgctccgggaccgggcttccgcctgggacagggacaccctcttcgccgtctttgggcccgacacggaaaacgcctcggagcacgccggggcggtgcaggtgggggacgccaagctcctcctcctccccgtgcggagcctctacggggtcttcgccctggccacgagcccctacctcctggagcgcttccgccgtgaggccctcatggcgggccttcaaccccctggggttcccgggctccgggaccccacccaggtcctcttggccccggggtcccgccttttgggagacggggagaaggtctacctcgaggacctggacctgaaggcccagggggaggagggggtcgccgcctgggagcggtggcttgccgagcgcaccgaggccccggtcctgggaaggctcgccgtggtgcacgacgacctcatgggcttcctcctggagaccgccacggaggtggtggcccgcatccgcctggacgacgagaccaagaccgtggccaagggggccctctggtacgaggagagcctccccgcggaaagcctcctctacgccctggtgcgggccgaccgctccttccgcaaggggaaggagcttcgcccagagggcgtctggggcctcttccggggcgtcttggaggagggcgggggggtgctccagcttgggggcaaggccaccgtgggccggggcctctgccgggtccgggtggggaggtag

*cmr5* (III-B):

gtgcgcacccgctcgcaggtgtgggcccagaaggcctacgagaaggtccgggaagcggccaagggcgagggccggggcgagtaccgggacatggccctaaagcttcccgtcctggtgcgtcaggcggggctttcccaggccctggccttcgtggactcccgggggaaggaggcccacaaggccctggggaaccacctggcccaggtgctgggctaccgggacctccgggagctggcggaggccgcccgggaggccgagctcctccagtacctccgcctcacccgggaggtcctggccgcggcggagtggttcaagcgcttcgcccaggccctcattgaggagtag

*cmr6* (III-B):

atgggccgcagatccgcgcttgagggcgtccgcctcccccaaggcaaggagccccaccggggcctctggctggacaagttcttgaggtcggcgaggcgggaggacaccgaggccaagcgggttctcgtccgggaagcggcggggatccccgagcccggggagtaccgggcgttttttgagcggtaccgcggggccctcgaggccctgggggccgaaatccgtgaggccaggaccctctcccgcctggtggtgggcctgggtggggaaggcgtgctggagacggccctcaccctgcaccgcgcctacggggtgccctacatcccgggctcggccctgaagggcctggcgagccgctacgcccacctttatctggagggcgaggcctggcggcgcgacctcgcccgcttccaccagggcgaggcccaggcggggcttttcggcaccacggaggagcagggcctcgtggtcttctgggatgccctcccccttcccgggaagtggaagctccaccccgacattctgaacccccaccaccctgactactacgggagcggggaggccccccctgcggactgggacagcccagtcccggtccccttcctctccgccacgggcaccttcctcctcgccctctccccggcccccggggtttctcccgaggaggccgggccctggcttcgggccgcctggcgcatcctggcctgggccctgcgggaggagggggtgggggccaagacctcctcggggtacgggcgcatagccctggaggagcctgcgtcccaaggggaaaagcccctggcgccggggccgagcccggtccttcaggacctcctcacccgggcccgcgccctctcctacagggaggtgccccgcttcctggcctcccaggcggaggccatcctgggcctctcggtggaggaggcccaggcccttcgccgggccctggaggagcggggcttcctccgcaacccccaggacctgaagcgctggcgcaaggagcatcccggcctggaaggggttctcgccaagctgggcctctcggcctag

CRISPR-3:

gttgcaccggcccgaaagggccggtgaggattgaaacccggaggaggtcataggtgagaaggccgtcttagagggttgcaccggcccgaaagggccggtgaggattgaaaccgccgccaccacgaggagatcctcgccgagctccgcctgttgcaccggcccgaaagggccggtgaggattgaaactgcggcggggccctggtggaggacccactaggggggggttgcaccggcccgaaagggccggtgaggattgaaacgtggagggcctgcgggagatcgtccggcgcgccccgggttgcaccggcccgaaagggccggtgaggattgaaactatgggtggttctattgggtggttaggggcaactgggtgttgcaccggcccgaaagggccggtgaggattgaaacaggtcctcggcctggacctacaggcggagagcgcgttgcaccggcccgaaagggccggtgaggattgaaaccaccaggtgctccagctcattaagagccaggcggccaggttacaccggcccgaaagggccggtgaggattgaaac

*cas3* (I-C):

atggaccccaaagcccacacccctaacccccagggagcctggcacgccctcaaagaccacctcgaggccgtttcccgcctagccgggggctttgcccagcactttggcaccccaagtctggggcaggccctggccctccttcacgaccttgggaaggccactcgggatttccaggcctacctcaaggtcgcggcggagggaaaaaggacaaacagcgtgccccatgccgtctggggtgcggccttggcttacgtagccctgggctcagggcagcatgagggttgggaagccttcgggctacccgtgatgggccaccacgccggtctccccaagaggggtgaggcggccagcaaactcgcccaagccctgaaggaggaacccttcaaagaggtcgcggcgttcttgcgctcctcggagcttcgcaccaggttagaaggcctgcttaatgaggccctaaaagaggtgcgctccaaaaccgcaggggaccctcttcgcctagactttctggtccgtatggccttctccgcccttgtggatgcggactatctagataccgaagcccactttgatcccagcgttgcccgcctgcgccaggaaggctactccctagaaaagctatgggcgagattccaccgcgaccaggaagcccttctccaaagggtacgtccttctcttgttaactcggtgcgccgggaggtctacgaggcctgcctgcaggcggcggagctttccccaggcctcttccgcctcaccgttcccacgggtgggggtaagacccggagcggccttgccttcgccctgaggcacgctttgaagcacgggctcaggcgggtggtggtggccatcccttacaccagcatcattgaccaaacggcccaggtctaccgggagatcttgggagaagaagccgtcctggagcaccactccgcctacgagcctcccctgggagaggaacaggaagaaaacgttctccgccagcgcctggccacggaaaactgggacgcccccctcgtcgtcaccaccaccgttcagctttttgaaagcctcttctccaaccgcccctctaagatgcgcaaggtccaccgactggcccgaagcgtcatcctcctggacgaagtgcaaacccttcccccggagcttttgaagcccaccctcgaggccctccgtctcctggccacccccgtggaggaggggggctacggggctaccgtggtcctctccaccgccacccagcccacgtttgaggtcgttcccaccttccagggccttcccgtccgggagatcgttccggattaccccaggcactttgctcgccttcagcgcgtggtttacgagcgccgcccgaagcccctttcctgggaagagctggccagggaactgcaagcgcgccctcaggtgatggtggtcctcaacacccgcaaggacgccttggccctcctcgaggccctgggggaggatccccacgcctaccatctctccaccttgctctgccccgcccaccgccgtgaggttctggaggaggtgcgaaggcgcttgcgagagggagcgccggtccgcttgataagcacccaggtggtggaagcgggggtggacctggacttcccggaggtctggagggctataggccccttggaccgggtggtccaggcggcggggcggtgcaaccgggagggccggctggacaagggaaaggtggtcctcttttggcctgaagagggcacaacccccaggggcccctaccgggtgggcgtggaaaaggcccgccttctccttgaggagcacccaccggagcggcttcacgaccccggtttgtaccaggcctacttcaaagagctcttcaccacggtcaaaaccgacaaggggatccaggagcaccgaaaggaccttgactaccccgaggtggctcggcggtaccgccttatccaggaggacacggtctccgtggtggttccctatggggaggggttggcgcgcctcgaggccttccagaaggccccctcactccagaactggcgacggctccaggcctacgtggtggggctcttccgcaaccaggtacgggagcggcgcggattcctcgaggccgtgccgggcttccaagacctctacgtctggcgtgggtcatatgacccgaggcggggactcgtggaggagtatgctgatcctagcgacctgatcgtctag

*cas5* (I-C):

gtggcgaggctcaaggtaaaggtttggggcgaatacgcctgtttctcccgccccgagttcaaggtggagcgggtttcctaccccgtgcccacccctagcgcagcccgcggccttttggaggccattttctggaaacccgagttccggtatgaggtgcggcgcatcggggtgctccgcctgggaacccccttcgccctcctgcgcaacgaggtaggaaaccgcatgggggcaaaacccttcttcgttgaggatgcgcgccagcagcggacaagcctcgtccttaaggacgtggcctacctggttgaggcggatatggtccttaggccccacgctaccgatcccctgcccaaatacctagagcagtttgaacggcgtctcaaaaaaggccagtaccaccacactccttacttgggcacgcgggagtttcccgcttacttctcgccacctgacggggaggtgcccgacgggggtttgaacctggacctagggcccatgctctttgatctcgcctttgtggaggacccaggccgtcccgagctcaccttcaagcgcccaggtagaggcgaggtccaggggtacgccctgccccttttctttcatgcccggatccgggaaggttggctggaggttcccgccgaaaagtaccaggagctctaccggctggaggaaggccatgctaagggagcttaa

*cas8* (I-C):

atgctaagggagcttaaggaggccttccaccgcttccgtaaagagggcgttctcctccctctcgcgtacaagaggaagagcgttgcctggttcatccgcctcgaggggggaaaggccaggctagaaggcccttacggaaagagggaaggggttgagcccatccctgcccccgaccgtcaacgttcgggaaaggccagcgaggccaacctgaagccctaccttctcgtggacgacgcccgctatgtgttgggcctctttgaagaggggagggaggatgaggccgcgctcctgcacaaagggtttaaggaacttaccgaaaaggcatggcgcgagacccagagcccgcacgtcaaggtagttctggacctgctctcgtctcctgagctcggggatcttcgggaggagtttcggcagaagggtgggaagccaagggacctcctcgccttcaaggtccacggcgtcttgcccacggagcttgaggaggtgcaggccttttgggctcgccacctcgctgaggagatccaagggggcaagggctattgcgccctctgcgggactttgggcccagtcttgcgcatcttccccagggaagtggtggttctgggccagaagtgccagctggtctccttcaaccagaaggcctttgagtccttcggcaaggagcaggccgcaaatgcccccctctgcttcacgtgcgcctcccaggtggtggacaccttggactacctgatccgctccgagcgccacagcaggcccctctaccgcgaggaagaagggggtctcaggaaccagcttgccgtcttctggcttagcgagaaagtggaagtacctcttgaaacgcccgtggacctggagaccattctgggggccgtgctgagcggggcacttgtccattccccgaccccacccgccgaccttacccaactccaggagctcctggccacgccttggacagggaaagcgtggcccctcaacctggatgaaacccgctttcaccttgccgtcctctccgccaacaaggggcggctggtggtgcgggagtggttggccgtctccctgagccgcctcaaggagaccttggggcgctttctcgagggcacccgcctcgttcgcccatggggggaagctccccaaccggtgcccgtaggggcgctagtccaagccttgggcgacggcaatcccggcttggtgagggggcttctccgcaccgcctacctcggcaccccccctccccatgccctttctttggcgatccaccttttgaggaaccccaagaccctcaccccggaggaaagggaaggttgggggcggcttcacgccctggtggcggcgctaaagctgtggttattttacggaaaggaggaggcaaagcgtatggccgagctggacaaggaaaggaagaacccggcctacctgtcgggccgcctcttggcggttctggaggaggcccagaagcgggcctcaagctacaccctcaggcggaccctggtggaccgcttttacggggcagcctccacgacccctgcggccactttcggggtccttctacggctctccaccaccgcccacctgcccaaggtgggccaagagctcaaccaggcggtggaggagatcctctctcgcctggacgaagccggcgggtttccccgcaccctgaacctggtcgggcaggcggagttcgccctgggcttctaccaccaacgggcccacttccgggcgaatcgggcgggcaagaagggagaaagcgcgcaaggagggtcatga

*cas7* (I-C):

atgagcgcgaggtatctagatcccaagaagcggcacgagttcgtcttcctgtttgatgtgagggacggaaatcccaacggggaccccgacgcgggcaacctgccgagggtggacccggagaccatgcacggcttggtcacggacgtggccctcaagcggaagatccgcgactacatcgccctaacccgcgaagggaagccccgtttcgccatcttcgtccagagcagggtcgcccttaaccggctcatcctcgaggggttccaggaggcgggcattgaacccgtgtccctccagctcgccgaggagcagatggccaacgagagcctgctggagcacctggacggcctggccgaggccggcttcagcagggaaggaggcaccctttattacagcggccaagccacgacggaaaaagagatcgccaagcttctgatgggggaggaggaagccgttgcccctttcaagaaggaactagaaaagctggctaaggagctcgccaaggccgtgaagggccgaaagatcaccgaagaggaccgggagagggcccaagccaagcttctggagcgcttttttgacatccgcatgttcggtgccgtcctcagcacggggctcaacgcgggccaggtgcggggtccggtccagctcacctttgcccggtccttggacccaatagcgcccctagaggtttccatcacccgggtggccattacccgggaggaggaccgggcccgaaaggaaacggagatgggccggaagcccttggtaccctacggcctctaccgggcccacggtttcttcaacccctttttggccgcgaagacgggggtacagccggaagatctagaagccctgtgggacgccttgcagcacctctttgagctggaccgctcggcggcccggggcgagatgacggtgcggggccttgccgtcttcagccacgaggacgcgaaaggcaacgctcccgcgcaccgtctcttcgggctcatccgggtggagcggcgggaaggggtggaggccccccgaagctttgccgactaccgggtccgggcgccgaaggaaggctcccttgaggcccacggttttcccggggtccaccttgcgtggctcgtccggccggagggcctcgaggacttaccgccccatgtgggatga

*cas4* (I-C)：

atgtgggatgaatccatccccatttccgccctccagcacttcgtctactgccccaggcagtgtgcgctcatccacctagaaggagtctgggaggataacctctacacccttcggggtcgccgggcccacgaagggttggacctgccggaagggctggtgcgggaaggggtgagggtagagtacgccctcccccttttctccgagcgcttaggcctagtgggccgggccgacgtggtggagttccacggcggagtgccctacccggtggagcacaaggtgggcccccggcgggcacgcaaggcggacgaggctacctccgcgcaaagcggcgaaggccgtggtggagcttga

CRISPR-4:

cggcccgaaagggccggtgaggattgaaaccccgcgcacccctccacggggcatagcaggtcatgttgcatcggcccgaaagggccggtgaggattgaaacggggcgggccgccatatctgtacccgctctagagcaactggagttgcatcggcccgaaagggccggtgaggattgaaaccgggaggaggcgctggaggtgcggagctacgaggcgttgcaccggcccgaaagggccggtgaggattgaaacccctcagggtccgttacttccacaaaagttttaaagtggttgcaccggcccgaaagggccggtgaggattgaaacgcacagctttcggacggtggctggcgaaacccccaactcgttgcaccggcccgaaagggccggtgaggattgaaatatgcccaagaggttgagcacgctgcacctccttgccgatgttgcaccggcctgaaagggccggtgaggattgaaacccgcccggtccctggagcaccagcctgtcccacgttgcaccggcccgaaagggccggtgaggattgaaac

CRISPR-5:

gttgcaagggattgaaccccgtaaggggattgcgacggcccctgacgaggttcctaggtgaggtcggtctccttctgggttgcaagggattgaaccccgtaaggggattgcgaccccccgtgtgggaggtggacctggggggcctcgagatgttgcaagggattgaaccccgtaaggggattgcgacgctccgggtgggccccgtcctcctcgggcccgagaggagagttgcaagggattgaaccccgtaaggggattgcgactgatgctgatcattgctcacctcctgcccggaggtgggttgcaagggattgagccccgtaaggggattgcgacaaatgcggtcctggagtgggaatacaacgaagtttccgttcagttgcaagggattgagccccgtaaggggattgcgacacggtaccggcgaaggacttggccacaccttgaatgaccgtcatgttgcaagggattgagccccgtaaggggattgatac

CRISPR-6:

gttgcaaacctcgttagcctcgtagaggattgaaaccttctccagcacccgccgcagagctgtggtatactgagttgcaaacctcgttagcctcgtagaggattgaaacgtgtcggagaagttccagaggcggaagacgagatagggttgcaaacctcgttagcctcgtagaggattgaaacggggaccggagggggcaccgcatccggcccagggagagttgcaaacctcgttagcctcgtagaggattgaaacccgctaccccggcatggagtcctaccagcctccctcgttgcaaacctcgttagcctcgtagaggattgaaacctggcccgggacctcaacgtggccgtcctcgccatcgttgcaaacctcgttagcctcgtagaggattgaaacagggatgcagggcgcttggatcttggcggtggcggtgttgcaaacctcgttagcctcgtagaggattgaaac

*cas2* (I-B):

atgagggaactgtacctggtcatcgcctacgatacccccgatgaccgtcgccgggcacggcttgccaagctgctcaagggctttggcgaaaggcggcagtactccgtgtttgaagcccggttgacccgggagcagtgggcccacctcaagggcaagctggaagccctggtcaacaaggaggaggacgtcttggcggtgtacttcttgcccccggaggcggtgggacgcacctggcgcatcggccacgaggggttgaagcgcctcgaggaccccgacttcgtctag

*cas1* (I-B):

gtgggcgtagtctacgtcctggaaaacgaagcctacctttccaaggaggggggaaccctcaaggtgtcccgccgggctgggcgggaggtgctcctgcaaaaacccctcatcgcggtggaggagatcgtgatcctgggcaacgcggtggtaaccccagccctcctcaagcactgcgcccaagagggtgtcggtatccactacctctcccgcaccgggacttactacgcgggcctcacccgtaccccgtccaagaacgctcctgctcgggtagcccagttcaaagcccacctggagcccacgtggaagctcgccttggcccagcgcttcgttttggggaagatccgaaatggcctcgtcttcctaaggcgcaacggggccgagggctgggaagggctcaaggaagccctcttagaggccgagcgcgcccaagatgaggaagcgctaaggggcgcggagggtcgggccgccgacctctacttccgggcctttgcagagcttttgccggaagagttcgcctttggggagcggagccgccgcccgccccgggacccggccaacagcctcctctccctggcctacaccctcctcgccaagcagtgcgaaagcgccctcctcgtggccgggcttgacccctatgtgggctacctccacgaggtgcgctacggccgcccctcgttggcgttggacctgatggaagagttccggagcgtccttgcggactcggtggttctaagcctcctcaataaccgccgggtgaccctcgaggactttgacgacagcgaaggtttcccccgtctacggaaagaggcgtggcccaagttcttaagggcttgggaagagcggctaaacgagcgggtacagcaccctctcctgagaaagcgcctggcctatcgcgaaatcctcttagcccaagcccggatcctggtcaagcacctcctaggggagcttccccgctacgagccctttgcggtgcgatga

*cas4* (I-B):

atgggccatgggggagccgtggaatacctgcccctctccaaggtgaacacggtggtctactgcccccggcgcttctatctggagtacgtactgggggaagcgcacgccaaccaccacctgattgagggccactacctccacgaaagggcctacaccgagccgggggaggagagcgggctttgggtgtggtcggaccggcttgggcttctcggggtggtggatcggctggagtggcggcggggggaggcctgccccgtggagtacaaactgggccgggccaaggaagaggcctacctctccgatgccgtgcagcttgccgctcaggccctttgtctccgagaatccagagggatcgaggcccggaggggattcgtctactaccacaaaagccacacccgccgggaagtggttttcaccctagagctctttcgcgccgtggaggcggctgtagtccgaatgcgggcccttctccgaagccctcgccccccaagggtagaggttcccccctccaagtgcgaggggtgtagcgtgcggggcgcctgccagcccgagctgtggcggaagggggtggcggggtgggcgtag

*cas8* (I-B):

atggagcgggcgatggtacaagcttctaccgtggctagggccaagcggctcgtggccgacgtgcgtttcggcgaagctcctagggaagccctcttggcctataaagaagcgcttgacgagccggagtttgcggccttcctggaacggatagatccacggatcgtacgctacctgcgcgtggcgcctgggggttcgcaggccctctctgatcccaagggcaaggacctgctcctgtacctccatcccttgcccggtgagcgccagatgcgggctgcctacgaggtggcgattgagggcttcctggagcacctcgaggccaaaggctaccccgtggtggagcgaggtgcaggctgggtgaaggcgtacgtgagccccaaggccccgtccttggacctcgagggggcctggaaggcctacatagaggaagcctttagcctggaggggctttcggctaggcttcttccccttctgaactccgtgcggctagccgggcggggcatcagcgcccccaaggtgcccgtgcccaccctgggagcccgggacttcctcgccgcctggtacctcgccaacctgctttccgtcaaagagcgcctagcctggcgggatcaggaaatacgccgcctagaggaggaggcttcccgccttcctgaaggttcggagaagtccaggaagctccgggagctggaaaagcggcggcaggaccaagagaaggagcttaagaaatacgggggggagcttcggaagaagtgggaagaaatcgtccgggaggaggagaaaagggccgaaaagcggcgcaagctggaagagcggctgcaaagggccaaacccaaggacaggccccgcttagagaacgagctccgggccctcgagcctcccctcgcccagtgggccctaaagggtctgggacaggccaaagacgaccccggacggctttggacgtggctggacccggaaagccccgaggctcccggggcgatccagaggcttaagccgtacctggggcgcttcggccctttggccaaagggcagctcaacaccgctgtgggcaacaagtttaccaagatcctcgaggagctcctccgcctcctcagcctttcgtcccccgaagtggaggtgccgcccctggtttccgaaacccccttcaccctggacctaagggatccgggggataaagccgatgtctgttacgggtgcgggcgccccctgggcaaggacaagctcaaggccagcaagctggtgttcgccagcccaagccagcgcctgcaaagcggaaatggtcaggaagagccctgggtctgcccttcctgcgccgcccttgccctcctttcccccatcaaacccggggaggggagcgtcttggtgcaggtaggaagctatggggctcccgaggcggccaagcacttcgcccgcctcctggttacgggaaccctgcacgtggcagccgggcgctacctgctcctaaatagcccacaggtgggggggaagcccttggcccaagctttgggccgcgtggtctacgccctccaggccttggggcaagaggtaaaccccaaggtcctggagcgctttcccttctacctggtggagggcgcgcaagaaatcccccttccccctagagcgctctggctttcccacgtcctgcaaagggcctttgcctcccggccggatgagggcggcgaggtcaaccgccccttgggggaggccttgcgctatgccctgggcgaccttccctggcatgccctgtacaccttggcgcggcgctatggccgggtggccgatcggttttcgctggaggatggcctgatgcggtatgcgagccttttggaaaaggaggtgggcatgaaggaaaacacggacctttcccaacgcttccgggacgtggccggcctcaccgggcttctcagcgcctgggtggggtacgtggagggccaggtgggccggaactcccaggaagccaaacgggcggtggtgaagcttctggataacctggagcgccctggggacttcctgtacgtggccgcctaccacctggacagcacccaggcccggctttacgaggcgggcggggctttcttctaccaggaggccaagcggctcctccaggaggcgggggccagggcgcaggaggcggaggaaggctcgggccgcttcctgaacgtctcccaggacgaccttcaccgggtttacgcgcacctggcggcgcgctatccgggcaaggcctgggaagggtttatctacgaggtgcgtctaagtctggcctcgaggttcccccagtacatccggatggaaaaggagggttag

*cas7* (I-B):

atggcgagcggactcacccccaaaggcgaggccctgtaccactgggacatctacgccctgctctacgccccccaggaggtctactttggccacgagacccaggtgaacgtgaacctcattgagaccgtaagcctccccgacgggagcgagcgggtttacctttcccccaccaagcgccgcggggtggagcggcgggccctcctttacgctcccgtggagcggaatgggcagcacctttacctcaccgacctcctgaactgcggcatccccaacacctgtggccgggagaactgccccgtctgccgggtctacggggccttggtcacggagcggcgggggaatatagaacgcaccactttcatcggccggctcacccacggaggtggcgtgtccatacccgcttggcagcccctggaaaagcagcgggccatgcacccttccgaccttaggcgggagtcgggcgaagagccccagcccttcaggcggcagtacggggctccgggcctcctcttccccgtgtacaaccacgtccttgccgcgagcgaaagggagttccgggcggtggcctacgccttcctggcaagccttcctcggctcggggcgggaaaccccaaagggctggacctctacgaggacgagaccttcggcccctacctggtcctggaccgctacaaggcccctttggggcggcgggtggtcctcccgcccacccttaaggaccccggggaggccctggcggagttccggcgcagggcggaggatgcccccgaaggagaagcgcttttccagcgccaccgggggagggcggccttggaggcgcttcggaggttggccaaggcttttgtggaggaggaccttccggctctggcttccgcttcctaa

*cas5* (I-B):

atgcacgccatcgccctcaggatccggcccacctcggccctcaccttccgggtgcttcccggctccatcctggacatcgccacctaccccttcgtcccgcccaccaccctttcgggatggctaaggcggctcttctgggcccagaagggcctccttcccccggatcagagggaggggaacaccccccgcttctacgtcctcccccggcgataccttccccttggggcgtaccccgtgggggagtggagggtccaccgcacccatcggcatgggccaagggccttcacccacagcgagttttcccgcctgcgccgggaaggcaagccccccaagggaggggaccttcagctgcatacttgggagtacctcctggccgaggagtttttgggggcggttttggcggagaaggaggaggacctccggcgcctccagggtcttgtgggctacggggccaagctgggcaaggagggcttcgcctacctcgaggccgtgggcgaacccctggaggtccgcctcgaggaaggggatgcgcccttcacccccgttcgtgcggaagcctggggcggggccatccagggggcttaccccctctaccgcttccgctttggggaggctgaagacccggaccccgcaagccccgaccccagtccggtcatagggtacgagggggcctactttgttctcccccgtgggcggggcctggccaaaggtttttccttggaagggcggtttttcgcttgggatctggtggagttcttatgggcgagggcatcgggagggtcttcttcctaa

*cas3* (I-B):

atgggcgagggcatcgggagggtcttcttcctaaaggacgaggtgggcttccagcccctttccaaccaccaggggctcgtggtcaagctcctggagtcgtggcgggaaggggatgagcccctggcgctttctccaaagaccaaggagcgcctcctccttgcggcgcggtaccacgacgacggaaagcgttttaccttccacatcgttccagacgggaagggcgggctcacctatagcttccgcgggcaccgcttccgggtggcccaggccgtccaagacccctacgcccaggcccttatccggggtcaccatgattactccacccgcgaggtggtgaacctggccgcggacttcctcgaggagggcttgggccaccgcttccccgaggaccttttcctcctcatgatggccgaccagttggaagcggagcttgcggtgcggttgtggcagaggcgggcaggggaggtccggcccttcgtggagtttgacctgttacccgacggcgaaggcgggttcctcttggacccgtggcccttccgggtggacgaagtcgccctggacttcctggtctacttccacccctaccggggcgaggaggccaaggtcgtggaggggtgggggcgggccttggtgggcgctttggaggaggggaaggtgcccgaggccttccgagaggaaaaacgccgggtgcgcctcaggccctgggcggccgcggcgaggaaagcggacgaccccgaggccttctacgcccggtttggcctgaagcccacccccttccaaagggaggtcttcaaggtagcggagggggaccccgcccacctcctcctcgcccccacggggacggggaagaccgaggccgcagccttccccgccctggcccggggggagcggctcgtcttcgtcctgcccacacggagcctcgtggacgacctggaggggcgctttcgccgctatctcaaaatcctggcccaggaggaagggcggcctaaggccttggtggtggacaccggccaccgccaggcgcgctttcgctttagcccagatggccaagaggaggccaccaaggagcggcacctctaccacgccgatgtcatcctcaccaccttggacaagctcctctaccgctacttcggctacgccgagggggtcaaatcctacacctttccccggcggatccacgaccggaggacgcttttcgtctttgacgaggtgcacctttacgaggccaccgcctgggtgaacttccgccacctgatcgcctccttgtacaaggcgggcgtgcgctttctggtcatgagcgccaccatgcccgggacgtaccgggaggagctgctcctcgagggcaccctggagcaccctgcggccaagcggccgagccgggtgctccgctacatgccccagggcaaccccatggaaatcatccaaagccaccggggcaagcgggttttggtggttctggaggaggtcaaggaggcggcggagctttacaagaggctcaaaggggagggggtctttctctaccatggccgcctggcggagggccaaaggagaagggtctttaggaaggtcaagcgccgggacaaggcccagaagccttacctcttgatcaccactcccgccatagaggtgggggtggacctggacgcagaggttctggtcacgaccctctgcccgccggaaaaccttctgcaacgcctgggaagggtgaaccgaaggggagggggccaagggaaggcctacgtggtgggggaaacctaccccgactacctcggaagcctgccggagggctacttggaactcctaaaggcgctggatgggcaagacctggcccaaggggaggaggagcgccttcggcaggccatccgctaccccaagtacctggaccccagggcggaaaccttctttgaggccctgcaggactacgtctacgggctggaccttacccaagaacccctccaccgcaagggcttcgtggccacccggggttggacgcccagcgtgcgcctccgccaaggagaggacgaggtggaggttcccatagaccggcttgtcggcaggaaggaggagctcactcccgtccgggttgtggagcggcttttgaccgatggagaaacgggcaaccggcgccgggaagaggttccattgcgctctggggagctttacggacgggagctggtgttggactacccctacccctacgacgcagagctgggctttgtggagttgcccaaggtgtttcagcgccttcgccatcccgacccccaacgggtccagcttctctacgccccggaaaaggacgcgggcaaggcgccggaaggcgttatggacaccgatcaggtaacgggcggggggagacgggttctctggtatctgggggagtcggcctgggcggaacccgccaagagcggggaggtggccgaagagaccgaggaagaggaagaagagggggactga

*cas6* (I-B):

atgcctcaggccgtggtcctcgagctcgtgggggaaaaaccccccctttaccccgcccgctatgcccacggcctcttctttgccctgctttcccgggtaagcccggagctggcccagaagctccacgaggctccgcgcaagcccttcaccctggcgccgcttcccagggctggccccgaaggggctaccttgaaggggaccctgaggctccgccttaccaccctggacgatggcctttttgcgccctttctgcgggccctcctggaggccgccccggatgggcttcccctgggggatagttcctaccggctggcgcgggtcctggctacccgcgaggggcaccccttggccggggccacctcctgggaggaactcaaggaggcccccaagcgggaaaaggtcacctttcgtttccttacccccacggtcttcgccacttctaagccgggtgggcgcacccgctacactcccttgcccgacccccgcctcatcgcaggctccctcctggacaagtggcaggctcacagcccctttccttacaacccaaaggaagaggccgccttgcgggggctttttgagttggacctcgaggtggcgggcttccgcaacctgcgcttccaccgggtacaggcggggaagggctttttcccgggcttcacaggtgagatgaccctgaggctttggagccagagcctcgaggcccgggaggccctggggcgcctccacgccctagccttcttcagcggcgtgggggctaagaccccctacggcatgggcctggcagtccccctctag

CRISPR-7:

gttgcaaacctcgttagcctcgtagaggattgaaactccggctcgtgaacggcgagattcagatcattcccggttgcaaacctcgttagcctcgtagaggattgaaaccatcacctcggcccgggccaggaccacccccacccgttgcaaacctcgttagcctcgtagaggattgaaacccgcctggctcgccggccccatcgcccgcctctggcaggttgcaaacctcgttagcctcgtagaggattgaaaccgctatggaccgaaacgactgcccactcgcgagcgcgttgcaaacctcgttagcctcgtagaggattgaaacaacacagcgtagaccgtaacggcccccagcgcgatgcgttgcaaacctcgttagcctcgtagaggattgaaacccgaaccgccgcctcagcgcggtgaggacctcgcccagttgcaaacctcgttagcctcgtagaggattgaaacgtgcggctcgtgaccaagtagccatggggtacttgttgcaaacctcgttagcctcgtagaggattgaaacttcggaacccgcctggagtacgccccatatcagcaggttgcaaacctcgttagcctcgtagaggattgaaaccgctgggcggggatcgcggcgagcgtggtcctcccgttgcaaacctcgttagcctcgtagaggattgaaactgtacgccctgcgaaactttgtgctccaaagcgtggtgttgcaaacctcgttagcctcgtagaggattgaaacagggcctcgaggcccttcaccaggaggccgaggcggcggttgcaaacctcgttagcctcgtagaggattgaaacctgagggagctggtaaacgagcttgggaacgcctggttgcaaacctcgttagcctcgtagaggattgaaacattcgccgcctcccagtagccgcgccaccacctcaggttgcaaacctctttagcctcgtagaggattgatac

CRISPR-8:

gttgcaagggattgagccccgtaaggggattgcgacctctttcaggatccacgcaaactcccttccttggggcttagttgcaagggattgagccccgtaaggggattgcgaccccgggcgagaggggcggcctcggggtccgacaaggcctccgttgcaagggattgagccccgtaaggggattgcgacgccgccccaagtccgaaaaacacgcttcttaacaccttacgttgcaagggattgagccccgtaaggggattgcgactttctggactgccttctggcctgagtgcgaggatcgccctcggttgcaagggattgagccccgtaaggggattgcgacacttcagcttacgcctcaaggctcacctccactaggggttgcaagggattgagccccgtaaggggattgcgacgattgcgacccagggaggagggccctggtttcgcctctttcaaatgttgcaagggattgagccccgtaaggggattgcgaccgtacgtgtagagggcttcttcgcccgtgtagagggcttctgttgcaagggattgagccccgtaaggggattgcgacttgaagtactgctcaagcttcttgccgaactcccctgccttgttgcaagggattgagccccgtaaggggattgcgacttgtaggcaacgggtttgtcctgggccttagtttcctgagttgcaagggattgagccccgtaaggggattgatac

CRISPR-9:

gttgcaagggattgagccccgtaaggggattgcgacctctttcaggatccacgcaaactcccttccttggggcttagttgcaagggattgagccccgtaaggggattgcgacgcctccaccatggggaggaggccctcctccctcgccacatacgttgcaagggattgagccccgtaaggggattg

CRISPR-10:

gttgcaagggattgagccccgtaaggggattgcgaccaaggatgacggtgcgagtcaacccgtccacttccgttgcaagggattgagccccgtaaggggattgcgacgccgatgaaggcggccagtacgcggctgtcgcttagttgcaagggattgagccccgtaaggggattgcgactggactgcctctacgagctccctgagtttcagcgcttccagccacttgttgcaagggattgagccccgtaaggggattgcgacaacctctccctgtcctgcaggacgcggaagaggttcagttgcaagggattgagccccgtaaggggattgcgacacgcgaacacgaccatagggagtgtcctcaaaccccacagttgcaagggattgagccccgtaaggggattgcgaccccaagaagctccaccagctccgccacgtcctcctcggttgcaagggattgagccccgtaaggggattgatac
